# Microwave-Assisted Synthesis of Quinazolines and Quinazolinones: An Overview

**DOI:** 10.3389/fchem.2020.580086

**Published:** 2020-11-16

**Authors:** Leyla Mohammadkhani, Majid M. Heravi

**Affiliations:** Department of Chemistry, School of Sciences, Alzahra University, Tehran, Iran

**Keywords:** quinazoline, quinazolinone, microwave irradiation, MWI, multicomponent reactions (MCRs), niementowski quinazoline synthesis

## Abstract

Microwave irradiation (MWI), as a unique, effective, sustainable, more economic, and greener source of energy compared to conventional heating, is applied in different organic transformations to result in the rapid formation of desired compounds due to thermal/kinetic effects. In this review, we try to underscore the applications of microwave irradiation (MWI) in the synthesis of quinazoline and quinazolinone derivatives that have been achieved and reported on in the last two decades.

## Introduction

In the past two decades, microwave irradiation (MWI) has become an effective, powerful, and green source of energy in the art of organic synthesis. As early as the 1950's, it was found that microwaves could heat water. The popular use of domestic microwave ovens happened during the1970 and 1980's as a result of actual and operative Japanese technology transfers and worldwide marketing. The use of MWI in chemical modification can, however, be traced back to the 1950's (Lidström et al., 2001; Bogdal et al., 2003; Kappe, 2004; de la Hoz et al., 2005). In 1986, Gedye et al. investigated four different types of organic reactions in a domestic microwave oven. They observed an increased rate of the formation of products compared to the rate of synthesis of the products under conventional reaction conditions (Gedye et al., 1986). Since then, the conduction of chemical reactions under MWI in the laboratory has started to gain wide recognition. However, MWI's role in organic synthesis has brought intense differences of opinion which resulted in diverse theories.

MW chemistry is the science of applying MWI to chemical reactions. When the rate of heat conduction is high between system areas, hot spots lacklong-term existence as the components reach thermal equilibrium rapidly. In a system where the heat transfer is slow, it would be possible to have the presence of a steady-state hot spot that may increase the rate of the chemical reaction within that hot spot. Based on this theory, specific molecules or certain functional groups within molecules should be excited when exposed to MWI. As a result, the time of distribution of thermal energy under MWI is much shorter than the period that thermal energy is distributed under ambient or conventional heating and laboratory conditions. The MW region of the electromagnetic spectrum lies between infra-red radiation and radio frequencies, relating to frequencies of 30 GHz to 300 MHz respectively. In order not to obstruct these uses, domestic MW ovens and industrial MW heaters are required to operate at either 2.45 GHz or 900 MHz, unless the apparatus is protected in such a way that no loss of radiation happens. Commonly, domestic MW ovens are designed and made to operate at 2.45 GHz (Mingos and Baghurst, 1991). Although synthetic chemists began to use domestic MW ovens in the mid-1980's, over the years, some problems have risen. The essential problem that one can have while using a commercial MW oven is caused by the nonhomogeneous distribution of energy. The other drawbacks involve the lack of temperature and pressure control, which does not allow for the use of flammable solvents and reproducibility (Bacher, 2016; Nain et al., 2019).

Consequently, special MW systems were designed for use in the laboratory, which demonstrates improved prospects for re-procreativity, fast synthesis, easy reaction optimization, and the potential exploration of new synthetic pathways. Dedicated microwave reactors allow fast heating of reaction mixtures to high pressures and temperatures–far above the boiling point of the used solvent. MWI can have certain advantages over conventional heating. Under MWI, reactions proceed to completion much faster, are performed under milder and greener reaction conditions, give higher chemical yields, require lower energy usage, and are selective or sometimes show different selectivity. Above all, its uses have become inevitable in cases when conventional approaches need constraining reaction conditions, when prolonged reaction times are required, or when certain selectivity is required. Thus, MW has been widely applied in the fields of organic and inorganic chemistry (Abramovitch, 1991; Loupy, 1993, 1994; Majetich and Hicks, 1994, 1995; Caddick, 1995; Strauss and Trainor, 1995).

Quinazoline and quinazolinone analogs are two important heterocyclic systems with diverse biological activities; thus, they have attracted much attention from synthetic organic chemists as desirable targets (Michael, 2004).

Quinazoline and quinazolinone derivatives constitute a noteworthy class of naturally occurring fused heterocycles (Wang et al., 1996; Sheu et al., 2000; Wu et al., 2001; Liu et al., 2018b), which were initially isolated from different natural sources such as plant families, microorganisms, and animals. Their structures were elucidated, showing diverse biological properties (Michael, 2003; Connolly et al., 2005; Mhaske and Argade, 2006; Khan et al., 2014, 2015; Kshirsagar, 2015). Several quinazoline and quinazolinone derivatives have passed clinical trials and are recognized as potential candidates for prescribed drugs (Inoue et al., 1976). Quinazoline and quinazolinone analogs possess a wide range of useful biological properties, including being antitumor (El-Azab et al., 2010; Sharma et al., 2013), anticancer (Wakeling et al., 2002; Vasdev et al., 2005; Chandregowda et al., 2009; Kabri et al., 2009; Pathania et al., 2014), anti-microbial (Gupta et al., 2008; Rohini et al., 2010; Rajasekaran et al., 2013), anti-bacterial (Mohammadi et al., 2014), anti-virus (Li et al., 1998), anti-inflammation, anti-tuberculosis (Nandy et al., 2006), and anti-obesity (Sasmal et al., 2012).

The first synthesis of the quinazoline nucleus was achieved and reported by Griess in 1869 through the reaction of anthranilic acid with cyanogen as a source of nitrogen (Griess, 1869). Von Niementowski optimized the reaction using amides instead of cyanogen (Von Niementowski, 1895). Owing to the diverse range of pharmacological activities, various synthetic routes for the synthesis of quinazolinone derivatives have been developed by employing 2-aminobenzoic acid, anthranilamide, anthranilonitrile, isatoic anhydride, 2-carbomethoxyphenyl isocyanate, *N*-arylnitrilium salts, and benzoxazines as starting materials (Michael, 2003; Connolly et al., 2005; Mhaske and Argade, 2006; Khan et al., 2014, 2015; Kshirsagar, 2015).

In 2007, a review on the synthesis of bioactive quinazolines and quinazolinones was published, focusing only on the synthesis of heterocyclic systems of specific biological activities or commercially important compounds (Besson and Chosson, 2007). In 2017, a review entitled “Green approaches toward [the] synthesis of substituted quinazolines” appeared in the chemical literature (Devi et al., 2017). Many researchers have used MWI as a green and sustainable source of energy in different organic transformations (Chatel and Varma, 2019). MWI has been frequently used in the synthesis of *N*-heterocyclic systems (Majumder et al., 2013). Applications of MWI as greener and more ecological trends emerge has also been extended to the synthesis of quinazoline and quinazolinone derivatives, particularly to those required for biological screening and in drug discovery studies, where rapid, high-yielding preparation of samples in highly pure forms are needed.

In the continuation of our interest in the chemistry of quinazolines and quinazilidinones (Heravi et al., 2008, 2009, 2010, 2011; Saeedi et al., 2011; Shiri et al., 2018) and our continuous attempts to conduct some of our reactions under MWI (Heravi et al., 1999, 2016; Valizadeh et al., 2010a,b, 2016; Fazeli et al., 2013; Heravi and Moghimi, 2013), in this review, we try to underscore all the successful strategies selected and progress made in the synthesis of quinazoline and quinazolinone derivatives, being conducted under MWI.

## Synthesis of Quinazoline Derivatives

### Intramolecular Heterocyclization

**Scheme 1:**
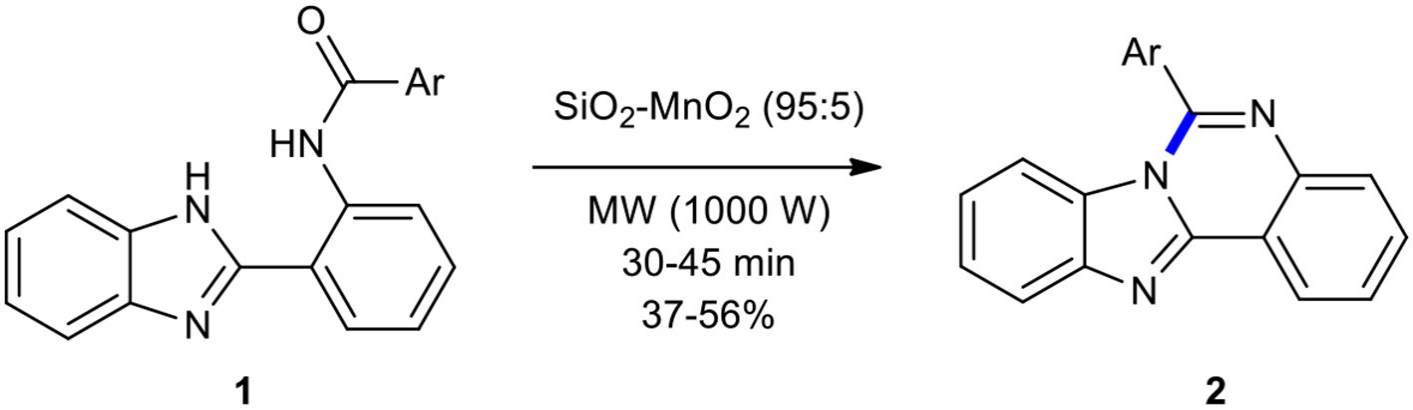
MW–assisted synthesis of **2** through intramolecular heterocyclization of **1**.

2-Benzimidazoylbenzamides have been employed as substrates for intramolecular heterocyclization to create quinazolines under MW conditions. For example, Pessoa-Mahana et al. reported the synthesis of 6-arylbenzimidazo[1,2-*c*]quinazolines **2** by intramolecular heterocyclization of 2-benzimidazoylbenzamides **1** under MWI in a solventless system (Pessoa-Mahana et al., 2004). Compounds **1** were efficiently and cleanly transformed into the corresponding tetracyclic compounds **2** in moderate yields under MWI in the presence of SiO_2_-MnO_2_ as a solid inorganic matrix in only 30–45 min (Scheme 1). The same reaction under conventional thermal conditions (anhydrous *m*-xylene, reflux) increased the reaction times. This novel and interesting strategy has shown the application of MWI in the heterocyclization for producing complex polycyclic systems.

### Intramolecular Friedel-Crafts-Type Cyclization

**Scheme 2:**
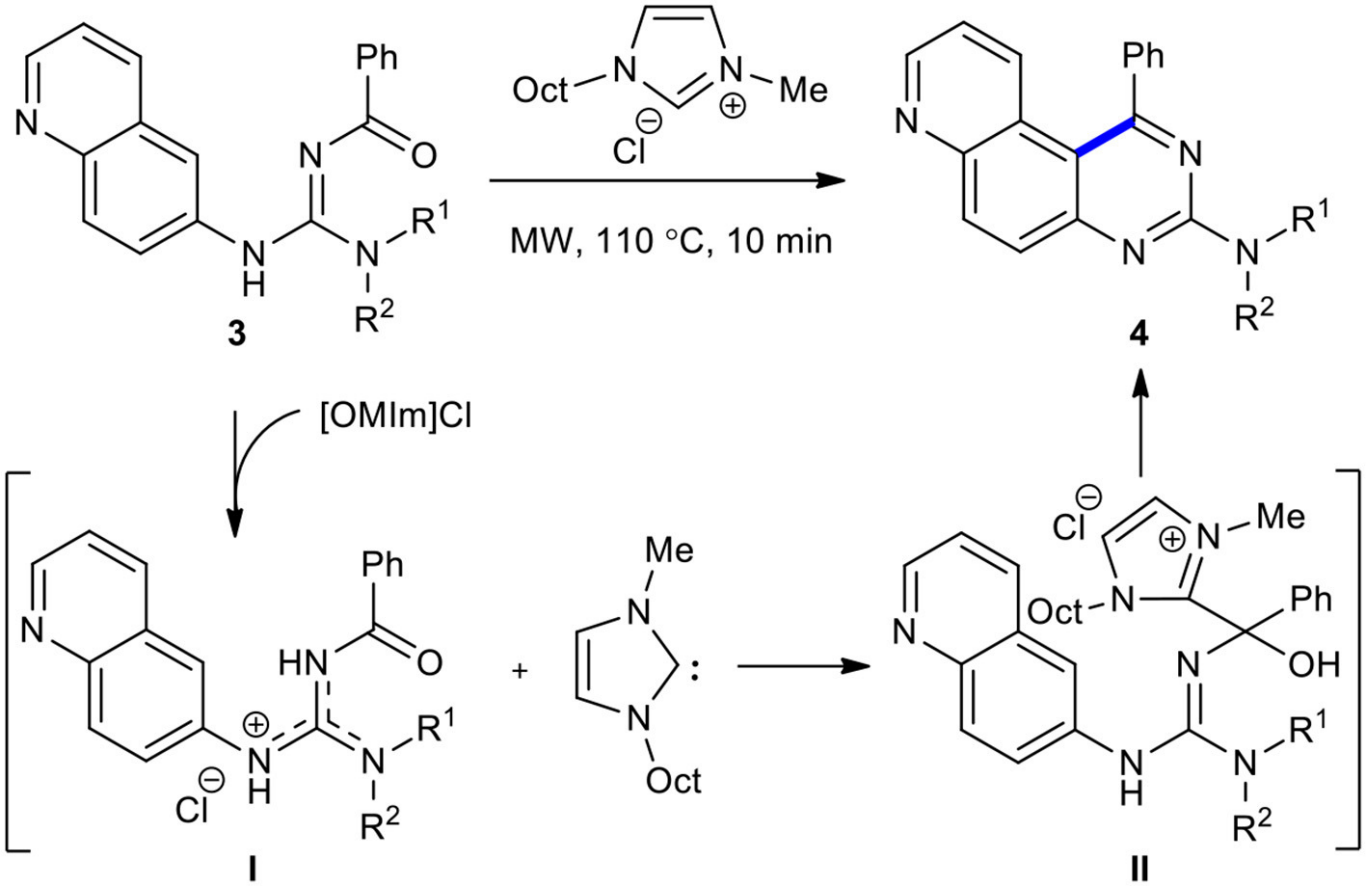
Mechanism of formation of **4** using ionic liquid as solvent under MWI.

Intramolecular Friedel-Crafts-type cyclization strategy has been applied in the synthesis of quinazolines. An original and efficient method based on the MW–assisted intramolecular Friedel-Crafts-type cyclization of guanidines **3** to access 4-phenylquinazolines **4** in [OMIm]X ionic liquid in 10 min was reported by Debray et al. (2010). The reaction mechanism includes three steps as represented in Scheme 2. In the first step, the basic arylguanidine abstracted the [OMIm] H-2 with the generation of the carbene and the acylguanidinium species (**I**). The second step involved the addition of the corresponding *N*-heterocyclic carbene to the hydroxyimino tautomer (**I**), providing the highly electrophilic intermediate (**II**). In the next step, the intermediate (**II**) underwent Friedel-Crafts-type cyclization along with the elimination of imidazolium and water, completing the synthesis of the expected products **4**. The use of traditional heating (110°C) instead of MWI required a longer time to achieve completion of this reaction (1 h instead of 10 min).

### Palladium-Catalyzed Annulation of *N*-Allylamidines

**Scheme 3:**
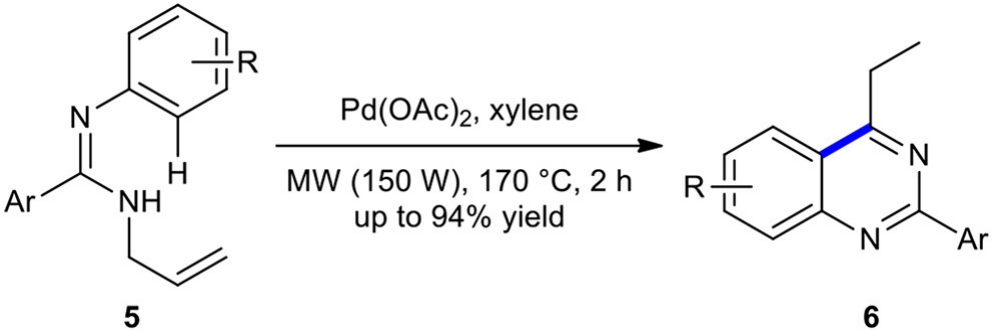
Pd(OAc)_2_-catalyzed annulation of **5** to create **6** under MWI.

*N*-Allylamidines have been used as substrates for palladium-catalyzed annulation to produce the quinazolines. For example, Pd(OAc)_2_-catalyzed annulation strategy for the construction of multi-substituted quinazolines **6** from *N*-allylamidines **5** under MWI has been developed and reported (Xu et al., 2015). Quinazolines **6** were obtained as the major products through the isomerization of Pd-alkene complex intermediate of *N*-allylamidines **5** and C–H activation in the presence of a catalytic amount of Pd(OAc)_2_ under MWI at 170°C in a sealed tube (Scheme 3). It was found that heating the reaction mixture conventionally improved the yield of the reaction.

### Cyclocondensation Using Various Methods

MWI has been used to promote cyclocondensation of various nitrogen-containing precursors with ortho-esters, benzaldehydes, alcohols, amines, ammonium formate, nitriles, HMTA, and thiourea, as described below.

#### Ortho-esters

**Scheme 4:**
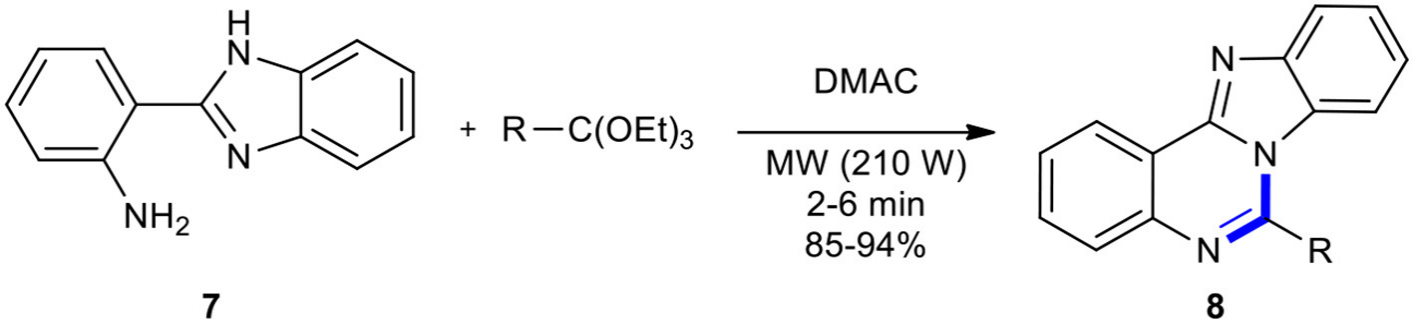
MW–assisted synthesis of **8** from **7** and ortho-esters.

Ortho-esters have been condensed with aniline derivatives under MWI to produce quinazolinones. As an example in this regard, the synthesis of 6-substituted benzimidazo[1,2-*c*]-quinazolines **8** was accomplished through the cyclocondensation of 2-(2-aminophenyl)benzimidazole (**7**) with ortho-esters in *N*,*N*-dimethyl acetamide (DMAC) using MWI and in the absence of the catalyst (Scheme 4) (Khajavi et al., 1999a). Products **8** were obtained, which showed a relatively higher yield compared to under classical heating. A comparison of two reaction conditions showed the emphasis on MWI as the main factor in improving the product yields in 2–6 min, which involved a prolonged reaction time. The reaction also occurred in the absence of a solvent.

**Scheme 5:**
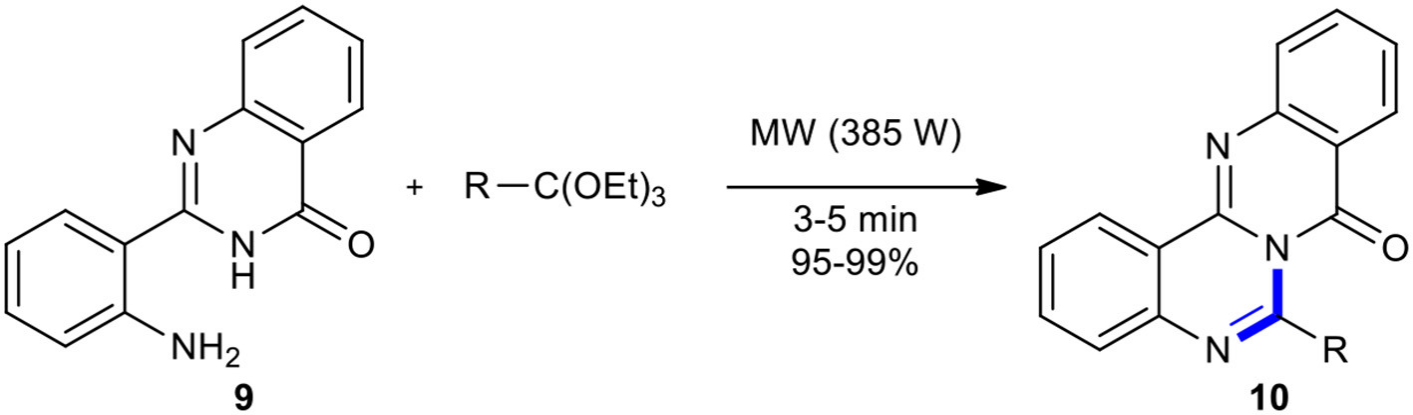
MW–assisted synthesis of **10** form **9** and ortho-esters.

Azizian et al. reported a strategy to rapidly prepare novel polyheterocyclic compounds **10**, 6-substituted quinazolino[4,3-*b*]quinazolin-8-ones, through fusing 2-(*o-*aminophenyl)-4(3*H*)-quinazolinone (**9**) with a range of ortho-esters under solvent-free MW conditions in the absence of organic or inorganic reagents (Scheme 5) (Azizian et al., 2004b). The strategy offered several advantages comprising cleaner reaction profiles, short reaction times, simple work-up procedures, and high yield of products, making it a useful tool for the synthesis of target products.

#### Benzaldehydes

**Scheme 6:**
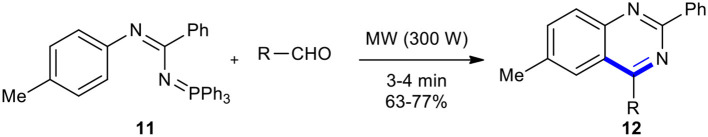
MW-promoted aza-Wittig synthesis of quinazolines **12**.

Benzaldehydes have also been condensed and cyclized with various amines under MW conditions to synthesize quinazolinones. For example, Kumar et al. accomplished the synthesis of biologically important quinazoline derivatives using a domestic microwave oven (Kumar et al., 2004). The advantage of this process is better yields, shorter reaction times, lower energy inputs, and an easy work-up procedure, as well as it being less expensive and high-yielding. The aza-Wittig reaction of *N*-imidoyliminophosphorane (**11**) with aldehyde derivatives under domestic MWI at 300 W for a period of 3–4 min led to the formation of the desired quinazolines **12** in good yields (Scheme 6).

**Scheme 7:**
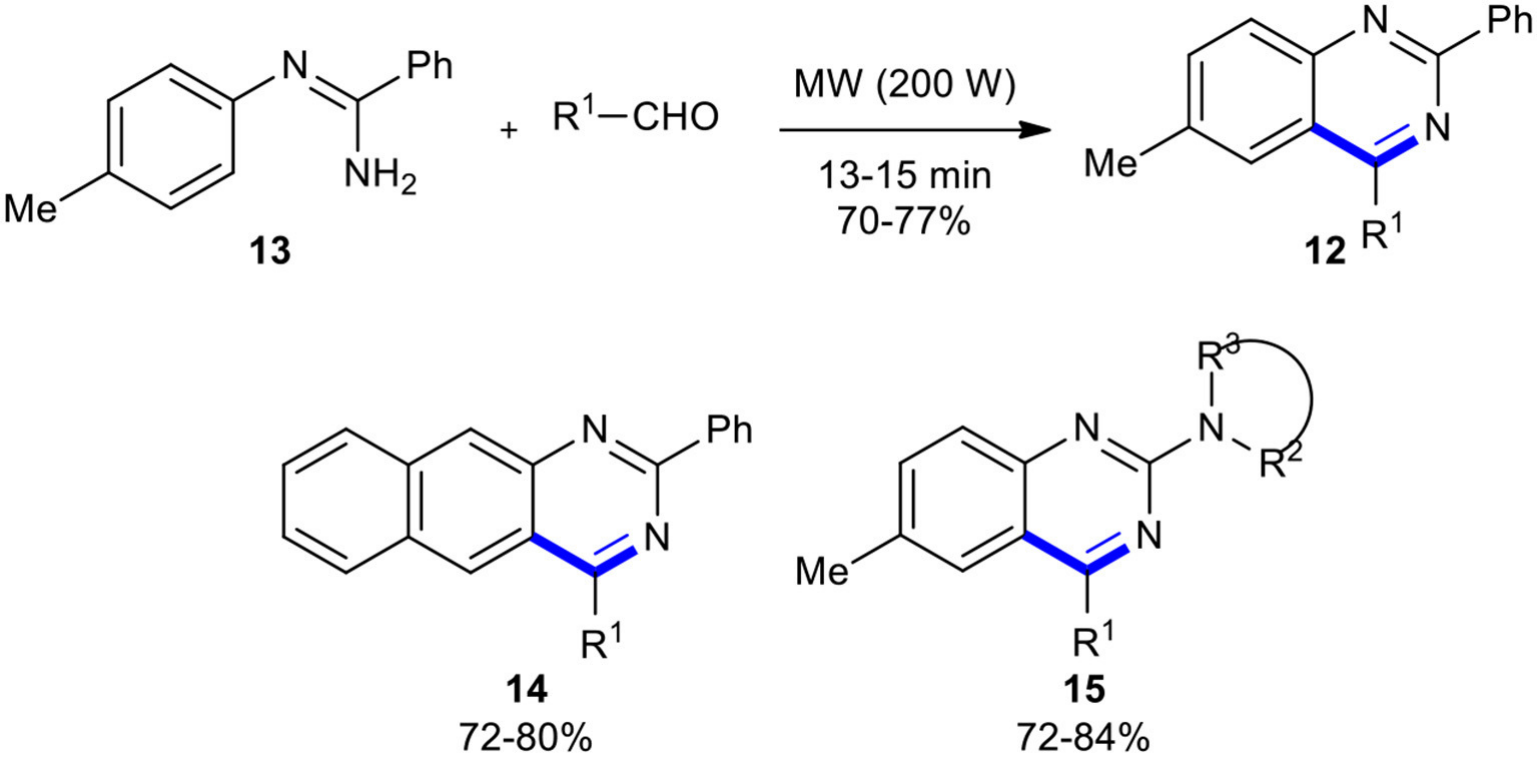
MW–assisted synthesis of **12**, **14**, and **15**.

Later, Kumar et al. synthesized quinazolines **12** *via* the MW–assisted direct condensation of *N*-arylamidine **13** with aldehydes, without their prior conversion to iminophosphoranes, in the absence of any Lewis acid catalyst (Kumar et al., 2005). This methodology could be employed to synthesize 2-secamino substituted quinazoline derivatives **14** and benzo[*g*]quinazolines **15** through the condensation of guanidines and *N*-naphthalen-1-yl-benzamidine with different aldehydes, respectively (Scheme 7).

**Scheme 8:**
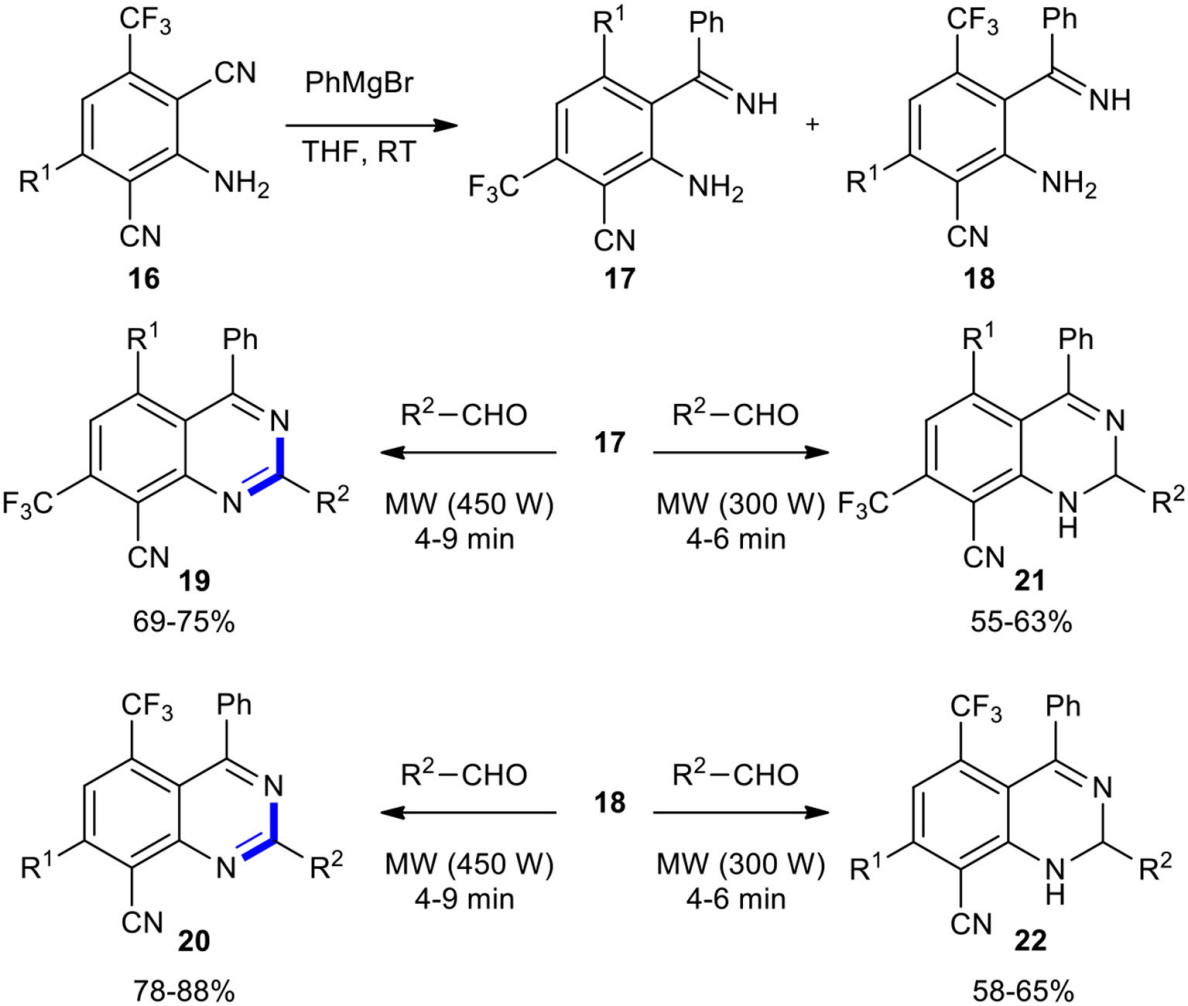
MW–assisted synthesis of quinazolines **19** and **20** from **17** and **18**, respectively.

A useful strategy to synthesize new quinazoline derivatives was developed by Maitraie et al. (2006). 2,6-Dicyanoaniline derivatives **16** on the reaction with Grignard reagents produced imine regioisomers **17** and **18**, which, after separation and identification, reacted with various aldehydes under MWI with 450 W to provide novel quinazolines **19** and **20**, respectively. Interestingly, when the same reaction was conducted under 300 W power of MWI, 2-dihydroquinazolines **21** and **22** were exclusively obtained (Scheme 8). Notably, quinazolines were obtained at a comparatively higher yield than the 1,2-dihydroquinazolines.

**Scheme 9:**
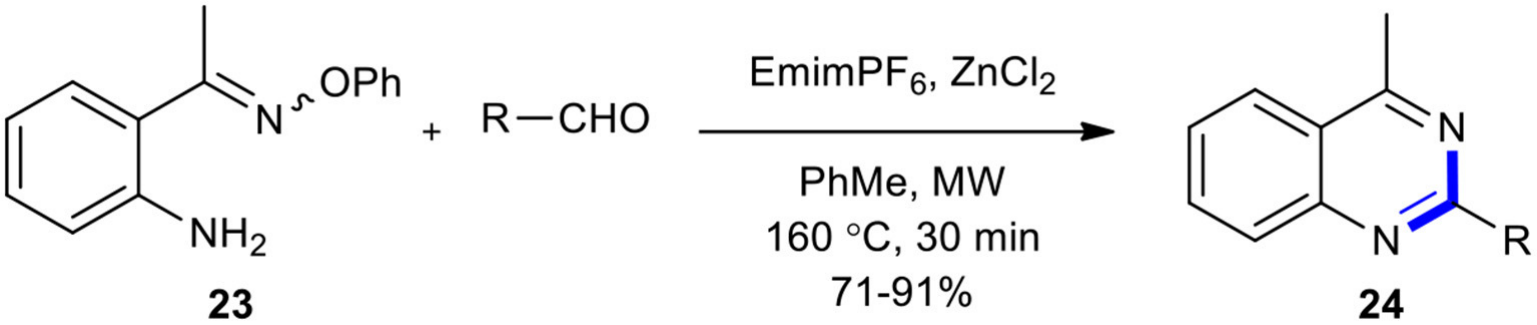
MW–assisted synthesis of **24** from **23** and aldehydes using emimPF_6_ and ZnCl_2_.

Portela-Cubillo et al. showed that MWI in the case of the reaction of 2-(aminoaryl)alkanone *O*-phenyl oxime (**23**) with aldehydes in the presence of emimPF_6_ as a catalyst and ZnCl_2_ as a co-catalyst in toluene is a suitable source of heating for the synthesis of quinazolines **24** in good to excellent yields (71–91%) (Scheme 9) (Portela-Cubillo et al., 2008). ZnCl_2_ in this reaction acted as a promoter which improved the yield of the products when less reactive carbonyl compounds were used. The notable features of this strategy involved mild reaction conditions, high yields, short reaction times, and no need for acids, bases, or metal catalysts. In addition, unlike other radical-mediated protocols, no initiators were required and no by-products contaminated the process.

#### Alcohols

**Scheme 10:**
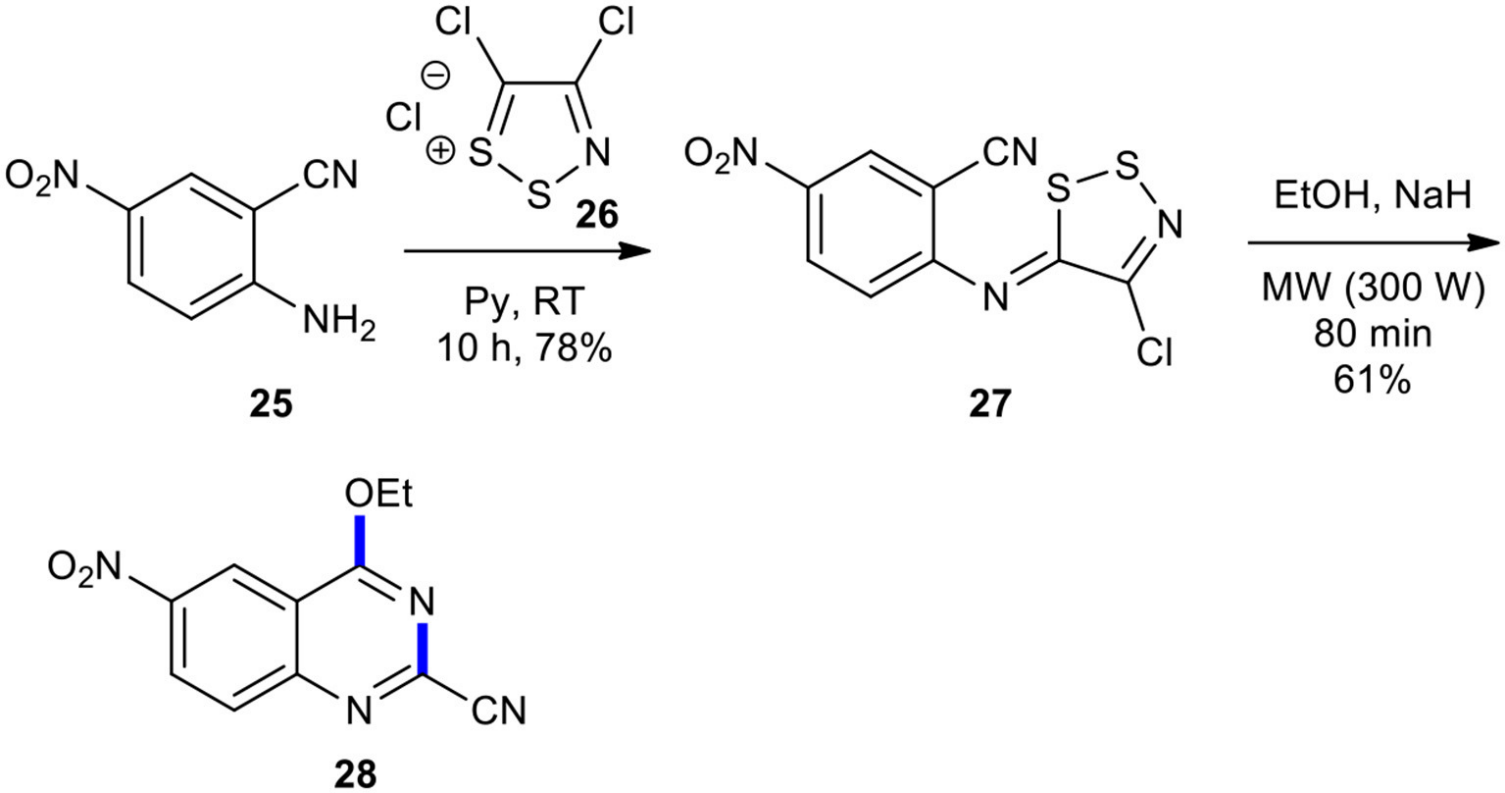
MW–assisted synthesis of **28** from **27**.

Condensation of alcohols with imino-1,2,3-dithiazole **27** to 4-alkoxyquinazoline using MWI has been reported. Besson et al. employed MWI for the synthesis of 4-alkoxyquinazoline **28** through the reaction of the imino-1,2,3-dithiazole **27**, prepared form aniline **25** and 4,5-dichloro-1,2,3-dithiazolium chloride (**26**), in the presence of sodium hydride in EtOH (Scheme 10) (Besson et al., 2000). Compound **28** was probably obtained *via* the addition of the alcohol to the cyano group, which was accompanied by cyclization and aromatization. Here, MWI was used as a powerful source of energy for accelerating the reactions and was cleaner compared to conventional heating (Besson and Rees, 1996; Besson et al., 2000).

#### Aliphatic and Aromatic Amines

**Scheme 11:**
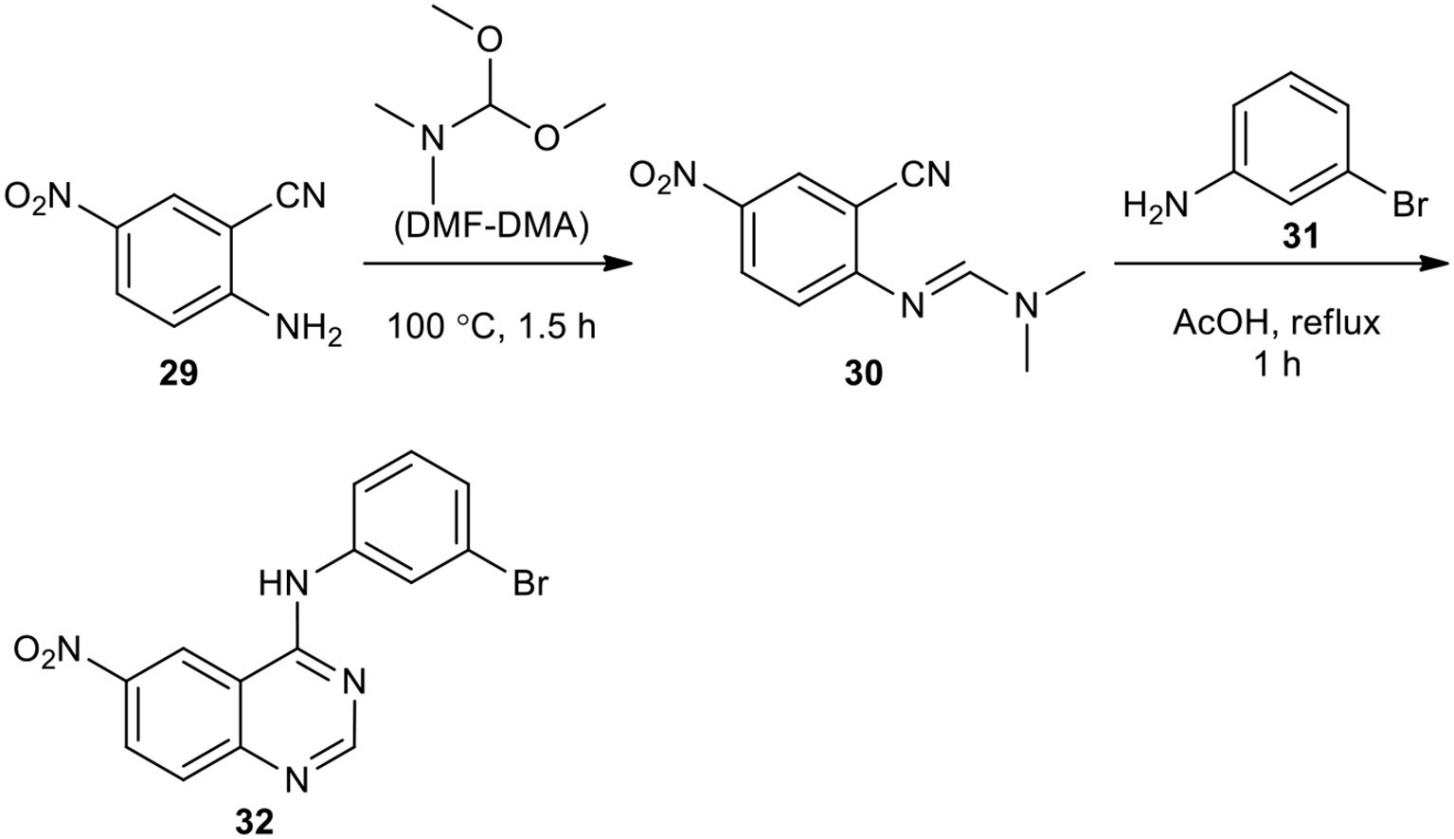
MW–assisted synthesis of **32** from **30** and **31**.

Aliphatic and aromatic amines have been employed as reactants to afford quinazolinones. Tsou et al. presented the synthesis of 6-nitro-4-(3-bromophenylamino)quinazoline (**32**) (Tsou et al., 2001). They reacted market purchasable 5-nitroanthranilonitrile (**29**) with *N,N*-dimethylformamide dimethyl acetal (DMF–DMA) as a reactant and solvent to give *N*-(2-cyano-4-nitrophenyl)-*N*,*N*-dimethylimidoformamide (**30**). The latter was then reacted with 3-bromoaniline (**31**) in AcOH under reflux to produce the desired compounds (**32**) (Scheme 11).

**Scheme 12:**
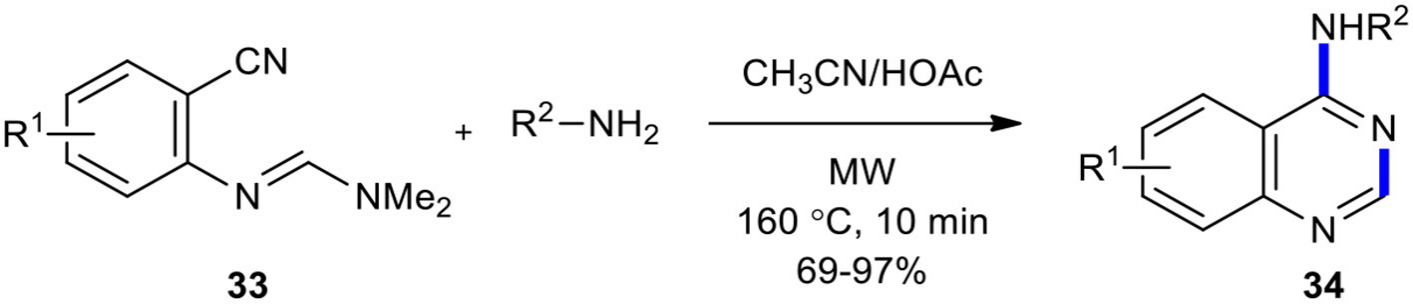
MW–assisted synthesis of 4-aminoquinazolines **34** from **33** and amines.

A simple, efficient, straightforward, and high-yielding strategy for the preparation of 4-aminoquinazoline derivatives **34** employing MW chemistry was reported (Yoon et al., 2004). The resulting products **34** were formed when the reaction between *N-*(2-cyanophenyl)-*N*,*N*-dimethylformamidine derivatives **33** and amines was conducted under the optimized reaction conditions (CH_3_CN/HOAc, MW, 160°C, 10 min) (Scheme 12).

**Scheme 13:**
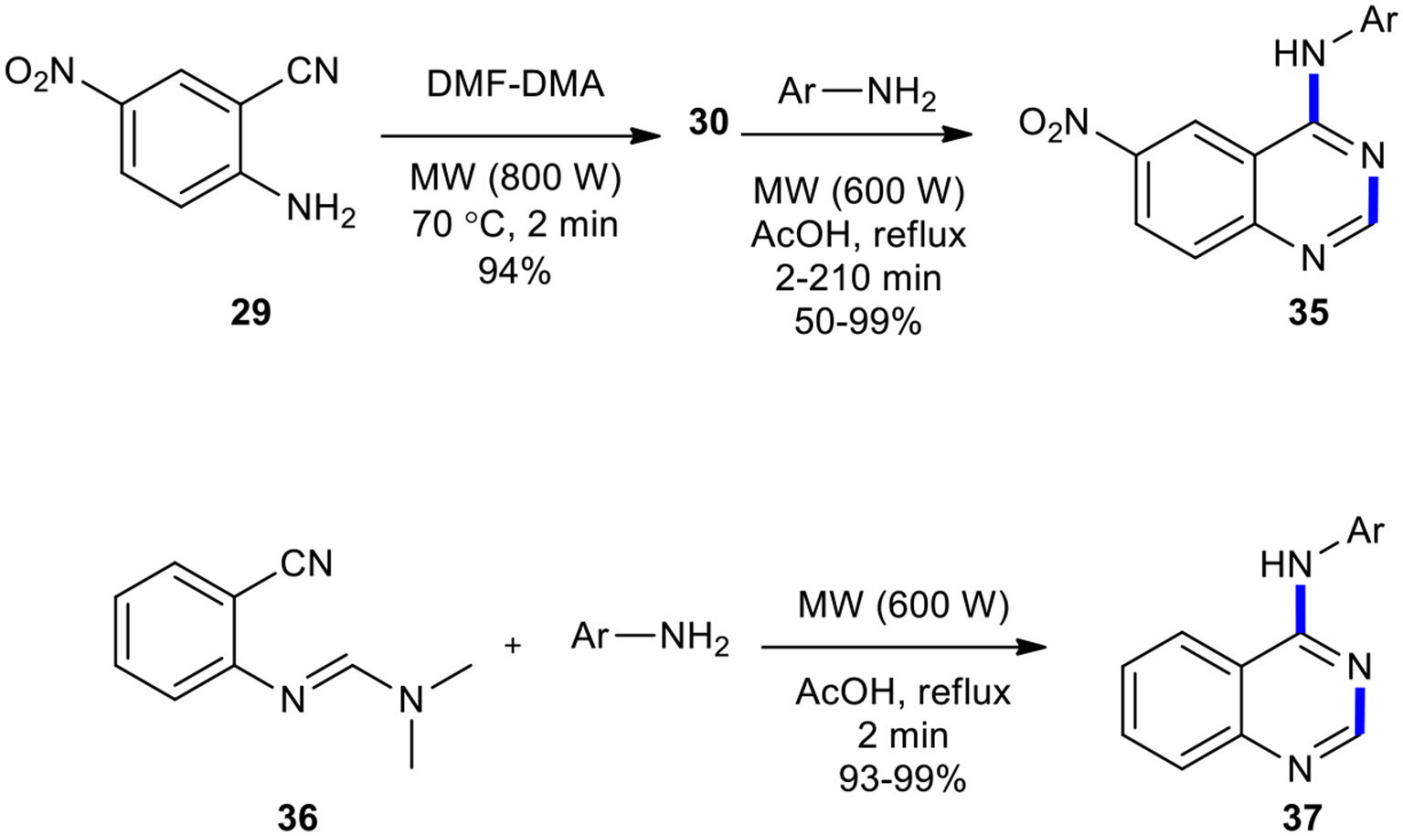
MW–assisted synthesis of **35** and **37** from **29** and **36**, respectively.

Foucourt et al. (2010) re-investigated the MW conditions of Tsou's reaction (Tsou et al., 2001) with the aim to define a well-established protocol, enabling a high level of reproducibility as well as easy scaling-up to a multi-gram scale. They synthesized 4-anilino-6-nitroquinazolines **35** from 5-nitroanthranilonitrile (**29**) on a multi-gram scale through the MW–assisted condensation and Dimroth rearrangement. The best result in the synthesis of the intermediate **30** was carried out in only 2 min under MWI at atmospheric pressure at 70°C, which gave the products in almost quantitative yields. They also synthesized 4-arylaminoquinazolines **37** (Scheme 13). In this MW heating, acetic acid reacted with anilines to generate the corresponding acetamides and gave cleaner products. This methodology was also used to access Azixa, a microtubule destabilizing agent and apoptosis inducer, in two steps in 64% overall yields.

**Scheme 14:**
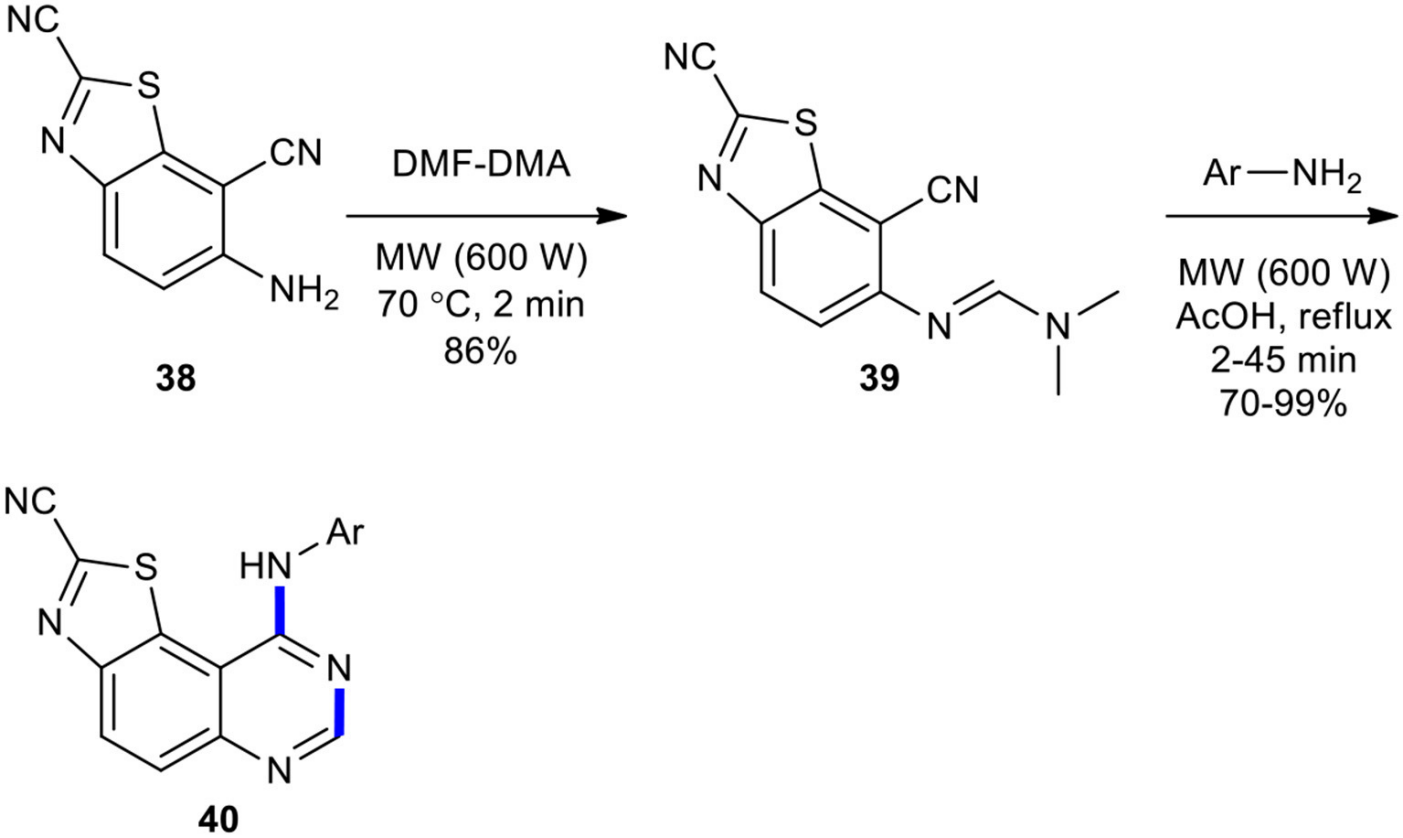
MW–assisted synthesis of **40** from **39** and anilines.

Foucourt et al. could also develop their strategy for the efficient and simple production of a forty molecule library of novel 6,6,5-tricyclic thiazolo[5,4-*f* ]-quinazoline scaffold as interesting DYRK1A inhibitors (Foucourt et al., 2014). The synthesis of the target molecules was started with 6-aminobenzo[*d*]thiazole-2,7-dicarbonitrile (**38**) which, upon reaction with DMF–DMA under MWI at 70°C, yielded (*E*)-*N*'-(2,7-dicyanobenzo[*d*]thiazol-6-yl)-*N,N*-dimethylformimidamide (**39**) in good yield (86%). Next, formimidamide **39** was cyclized to thiazolo[5,4-*f* ]quinazoline-2-carbonitriles **40**, the expected compounds, in 70–99% yields *via* MW–assisted thermal-sensitive Dimroth rearrangement using appropriate anilines in acetic acid at 118°C for 2–45 min (Scheme 14).

**Scheme 15:**
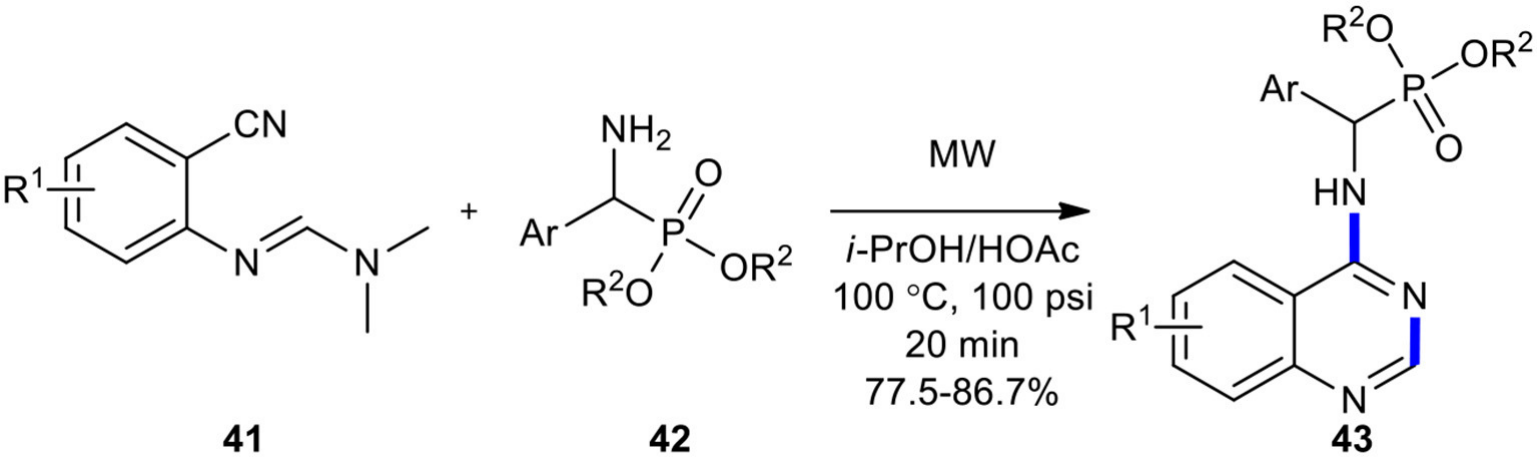
MW–assisted synthesis of **43** from **41** and **42**.

MW technology was employed as an efficient, mild, and environmentally friendly method to increase yields and shorten reaction times in the synthesis of (quinazolin-4-ylamino)methylphosphonates **43** (Luo et al., 2012). The desired products **43** were easily prepared in higher yields compared with a conventional mode of heating when *N*'-(substituted-2-cyanophenyl)-*N*,*N*-dimethylformamidines **41** was reacted with dialkyl amino (phenyl)methylphosphonates **42** under the optimized reaction conditions (MWI, *i*-PrOH/HOAc, 100°C, 20 min) (Scheme 15).

#### Ammonium Formate

**Scheme 16:**
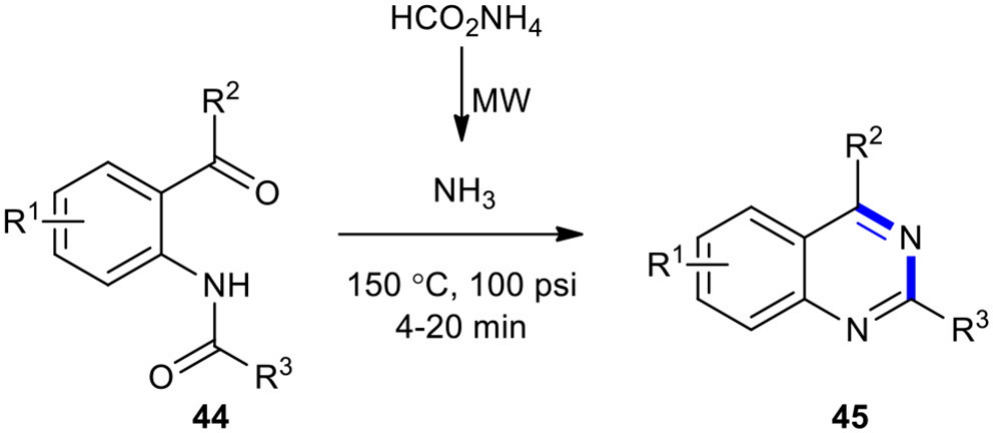
MW–assisted synthesis of **45** from **44** using ammonium formate.

Ammonium formate (HCOONH_4_) have been employed as substrates for the synthesis of quinazolinones under MW conditions. For example, 2,4-disubstituted quinazolines **45** were obtained upon treatment of acylamides **44** with HCOONH_4_ as a source of NH_3_ under MW activation at 150°C for 4–20 min (Ferrini et al., 2007). Heating of the reaction probably led to the decomposition of HCOONH_4_ and generation of the imine which was cyclized in the acid environment owing to the presence of excess HCOONH_4_. A temperature of 150°C and the presence of the acid afforded the requisite quinazoline **45** (Scheme 16).

#### Nitriles

**Scheme 17:**
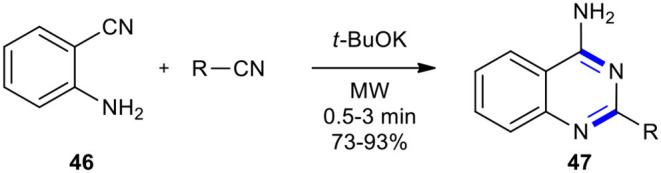
MW–assisted synthesis of **47** from **46** and nitriles.

Nitriles have been used as reactants in the synthesis of quinazolinone derivatives. A research group in the Universidad de Santiago de Compostela synthesized 4-aminoquinazolines **47** by the reaction of anthranilonitrile (**46**) with various aromatic nitriles in the presence of a catalytic amount of *t-*BuOK as a base in a domestic microwave oven as a heating device in the absence of solvent (Seijas et al., 2000). The products **47** were obtained in good to excellent yields and in a very short irradiation time (Scheme 17).

**Scheme 18:**
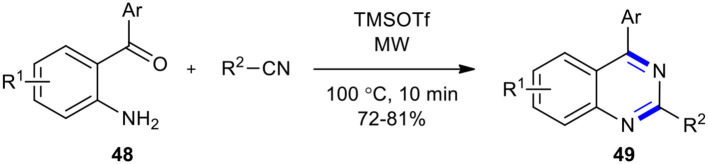
MW–assisted synthesis of **49** from **48** and nitriles.

A solvent-free, transition-metal-free, rapid, and efficient reaction to prepare 2,4-disubstituted quinazolines **49** *via* the Lewis-acid-catalyzed activation of nitriles and intramolecular cyclization in a one-pot reaction sequence was developed (Saikia et al., 2018). The reaction was performed in the presence of a catalytic amount of trimethylsilyltrifluoromethane sulfonate (TMSOTf) under MWI. Nitriles, employed as a nitrogen source and activated by TMSOTf, were reacted with 2-aminophenyl carbonyl compounds **48**. The best result was achieved when **48** was treated with nitrile and TMSOTf under MWI at reflux 100°C for 10 min (Scheme 18). The significant features of this approach included clean and mild reaction conditions, very short reaction times, and a broad substrate scope.

**Scheme 19:**
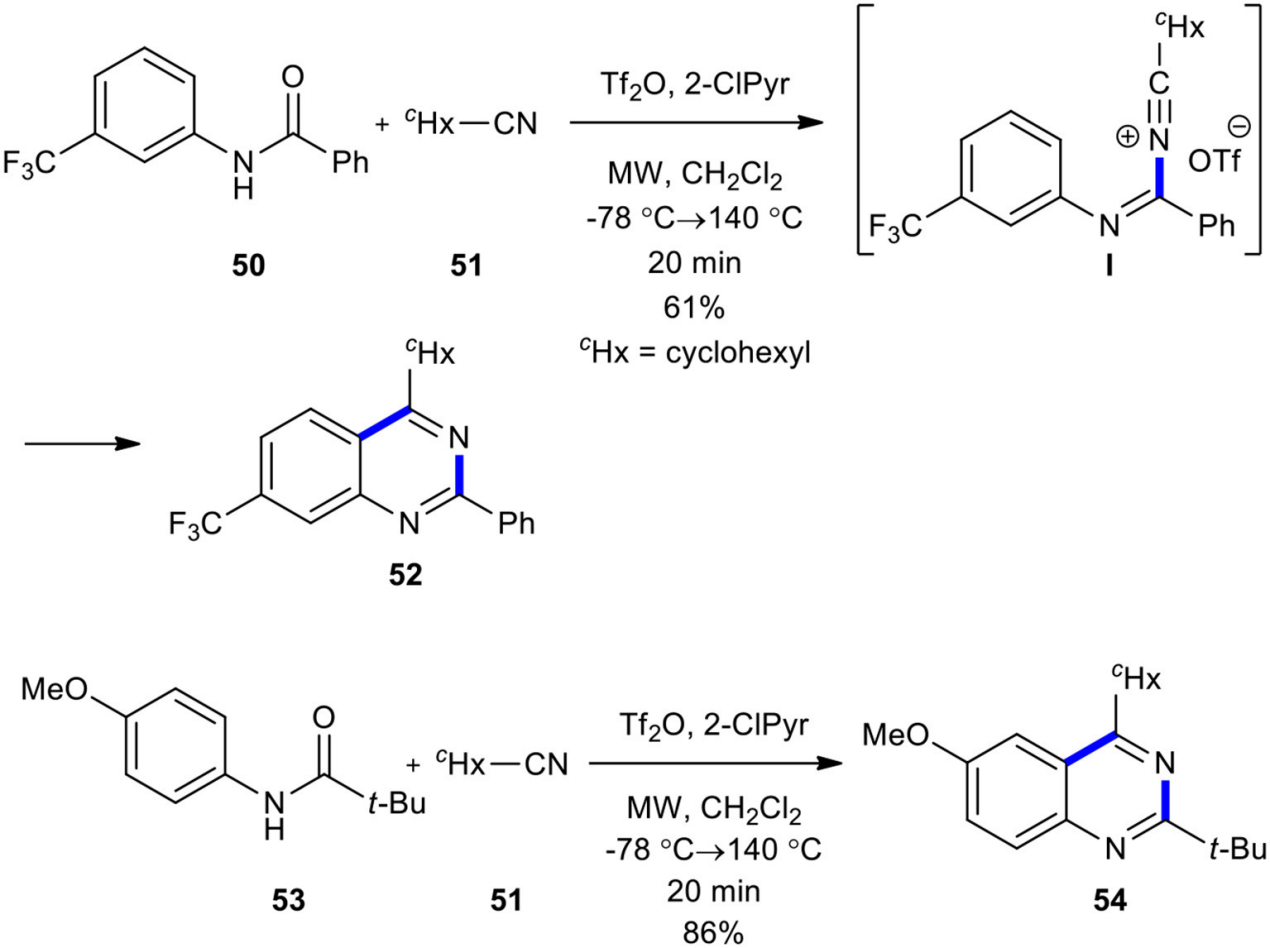
MW–assisted synthesis of **52** and **54** from **50** and **51**, respectively.

Movassaghi and Hill compared conventional heating and MWI in the condensation of amides with cyclohexanecarbonitrile as a nucleophile to construct an azaheterocycle scaffold (Hill and Movassaghi, 2008). The condensation of amide **50** with cyclohexanecarbonitrile (**51**) in the presence of trifluoromethanesulfonic anhydride (Tf_2_O) and 2-chloropyridine (2-ClPyr) in the sealed reaction vessel in CH_2_Cl_2_ under MWI heating at a lower temperature (−78°C) of 140°C for 20 min afforded the expected quinazoline **52** (Scheme 19). The above synthetic route could complete the conversion of the sterically hindered substrate, amide **53**, into the desired quinazoline **54**. This approach was found to be superior in comparison with heating in an oil bath. The MWI also shortened the reaction times and increased the yield of the products.

**Scheme 20:**
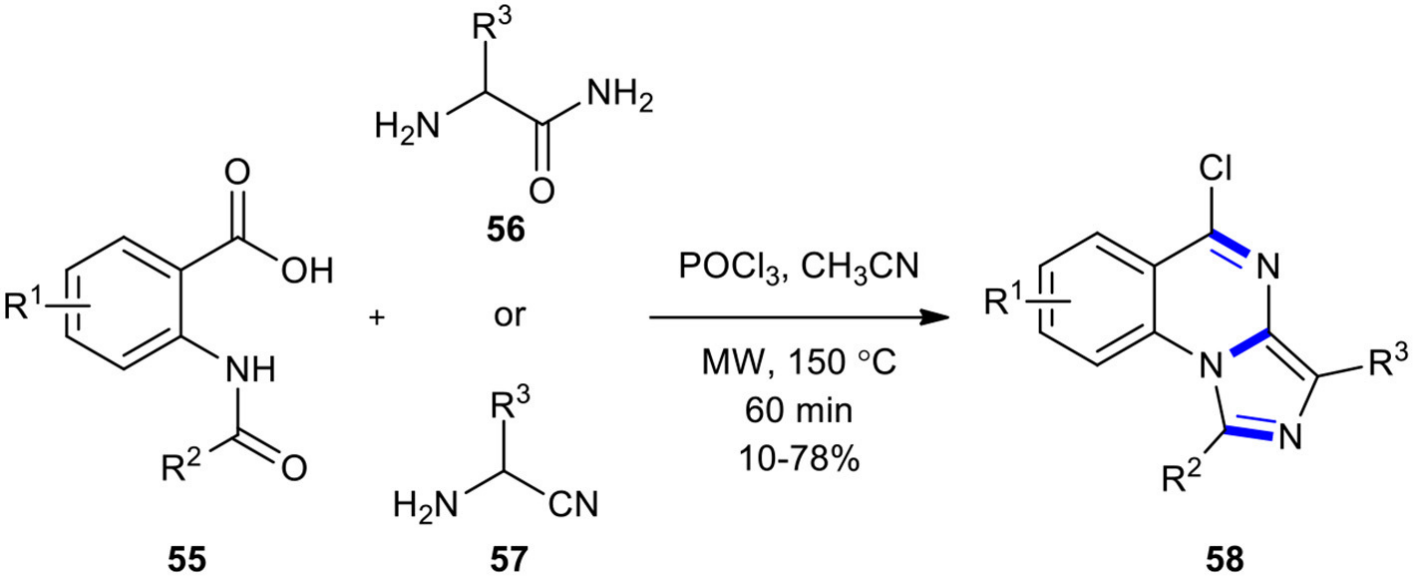
MW–assisted synthesis of **58** from **55** and **56** (or **57**) using POCl_3_ as condensing reagent.

The one-step synthesis of a new tricyclic product, 5-chloroimidazo[1,5-*a*]quinazolines **58**, was reported by Li et al. *via* the reaction between *N*-acylanthranilic acids **55** and 2-amino acetamides **56** (or 2-amino-acetonitriles **57**) in the presence of phosphorus oxychloride (POCl_3_) as a condensing reagent under MWI (Scheme 20) (Li et al., 2009). The introduction of EWG at the 5-position of 2-acetamidobenzoic acid improved the yield of the expected products.

#### HMTA

**Scheme 21:**
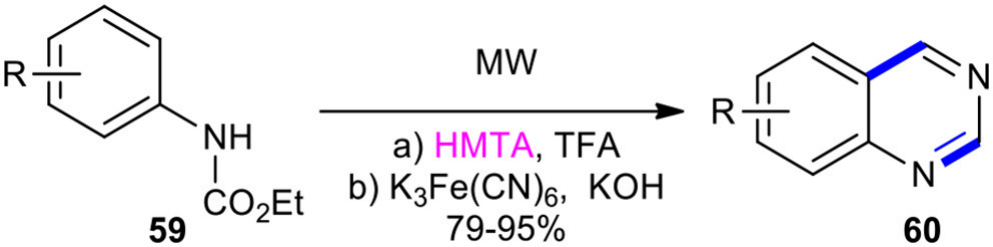
MW–assisted synthesis of **60** from **59** and HMTA.

Hexamethylenetetramine (HMTA) has been condensed with ethyl phenylcarbamates under MW conditions to synthesize the quinazolinones. As an example, Chilin et al. established the benefits of using MWI in the one-pot synthesis of the quinazoline scaffold as a high-yielding and user-friendly protocol. This method used ethyl phenylcarbamates **59** as a simple and easily available starting material (Chilin et al., 2007). The reaction of compounds **59** with HMTA in trifluoroacetone (TFA) to carry out aminomethylation and intramolecular cyclization furnished the dihydropyrimidine ring, which was subjected to oxidative dehydrogenation upon exposure to K_3_Fe(CN)_6_ in aqueous ethanolic KOH to obtain the desired quinazolines **60** (Scheme 21).

#### Thiourea

**Scheme 22:**
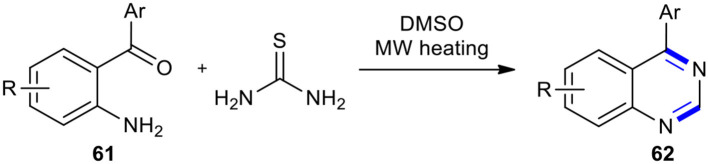
MW–assisted synthesis of **62** from **61** using thiourea in dimethyl sulfoxide.

Thiourea has been used as an efficient reagent for condensation with 2-aminobenzophenones to create the 4-aryl substituted quinazoline derivatives. A very simple and facile synthetic route for the direct conversion of substituted 2-aminobenzophenones **61** into 4-substituted quinazolines **62** under MWI in the presence of thiourea in dimethyl sulfoxide (DMSO) as a solvent and reagent was reported (Wang et al., 2016). The reaction was carried out through thermal decomposition of thiourea to generate carbodiimide and hydrogen sulfide, which were, respectively, reacted with 2-aminobenzophenone and DMSO to furnish 4-phenylquinazolin-2(1*H*)-imine intermediate and methanethiol (or other sulfur-containing molecules) as a complementary reducing agent. The reaction of 4-phenylquinazolin-2(1*H*)-imine intermediate with the sulfur-containing reducing agents formed 4-phenyl-1,2-dihydroquinazolin-2-amine which was subjected to the elimination of ammonia to complete the synthesis of substituted quinazoline derivatives **62** (Scheme 22).

**Scheme 23:**
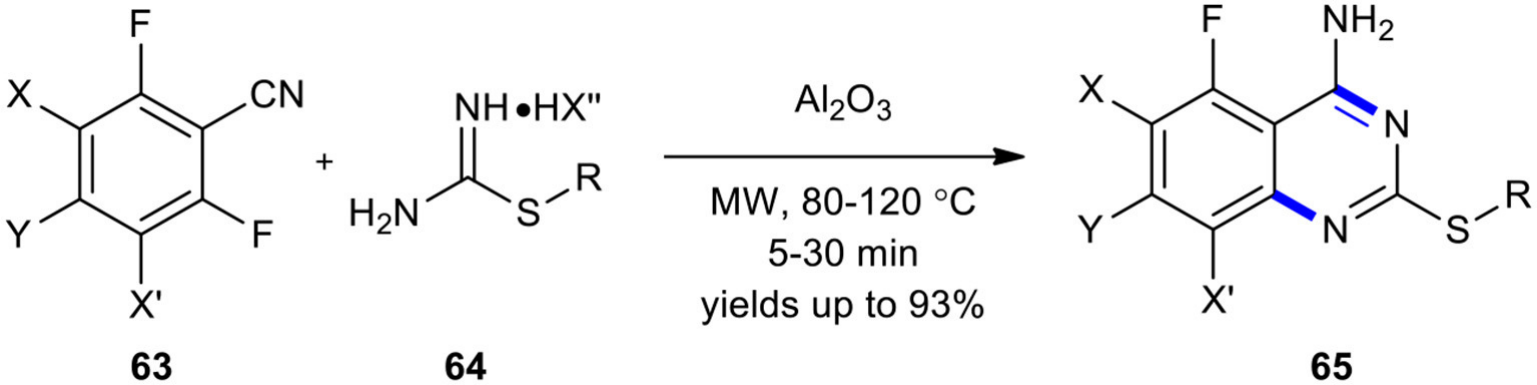
MW–assisted cyclization of **63** with **64** using basic alumina to synthesize **65**.

An inexpensive, environmentally benign, and efficient protocol was developed for the synthesis of the fluorinated 2-alkylthio-4-aminoquinazoline scaffold, with a broader sulfur-containing substrate scope, that combined the use of MWI and basic alumina, acting as the solid support agented base promoter (Liu et al., 2018a). The synthesis of 2-alkylthio-4-aminoquinazolines **65** was achieved by MW–assisted cyclization of *o*-fluorobenzonitriles **63** with *S*-alkyl isothiouronium salts **64** and basic alumina at 80 or 120°C for 5–30 min (Scheme 23). The use of MWI could considerably improve product yields.

### Three-Component Reaction

Multicomponent reaction (MCR) is an attractive synthetic strategy where three or more substrates combine to form a single product in a one-pot fashion. MCR is a useful and powerful tool that has been used for the rapid and efficient synthesis of various complex molecules and natural products (Armstrong et al., 1996; Toure and Hall, 2009). Moreover, it is characterized by its environmental friendliness, atom economy, high yields, time efficiency, and low waste production. As a type of MCR, one-pot condensation of aminobenzonitrile (or anthranilic acid and its derivatives), ortho-esters (or formic acid), and amines under MWI is one of the most straightforward procedures for the construction of quinazoline and quinazolinone derivatives.

**Scheme 24:**
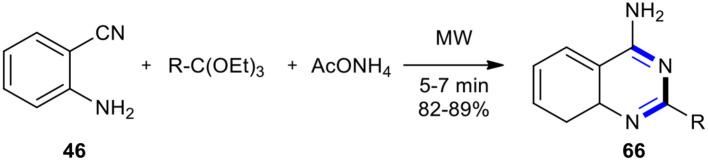
MW–assisted MCR of **46**, ortho-esters, and AcONH_4_ to produce **66**.

Rad-Moghadam and Samavi used MW technology to develop a facile and rapid method for the synthesis of 4-aminoquinazolines (Rad-Moghadam and Samavi, 2006). The synthesis was accomplished in a few minutes through a one-pot multi-component reaction between 2-aminobenzonitrile (**46**), ortho-esters, and ammonium acetate (AcONH_4_) under solvent-free and MW conditions, leading to the desired products **66**, 2-alkyl-4-aminoquinazolines, in good yields (Scheme 24). AcONH_4_ was employed as a separate synthon for the first time for *N*-3 in the synthesis of 4-aminoquinazolines. The results were compared with that of a multi-component reaction under solvent-free conditions. MW conditions with conventional heating (refluxing absolute ethanol) showed a preference for the first system.

**Scheme 25:**
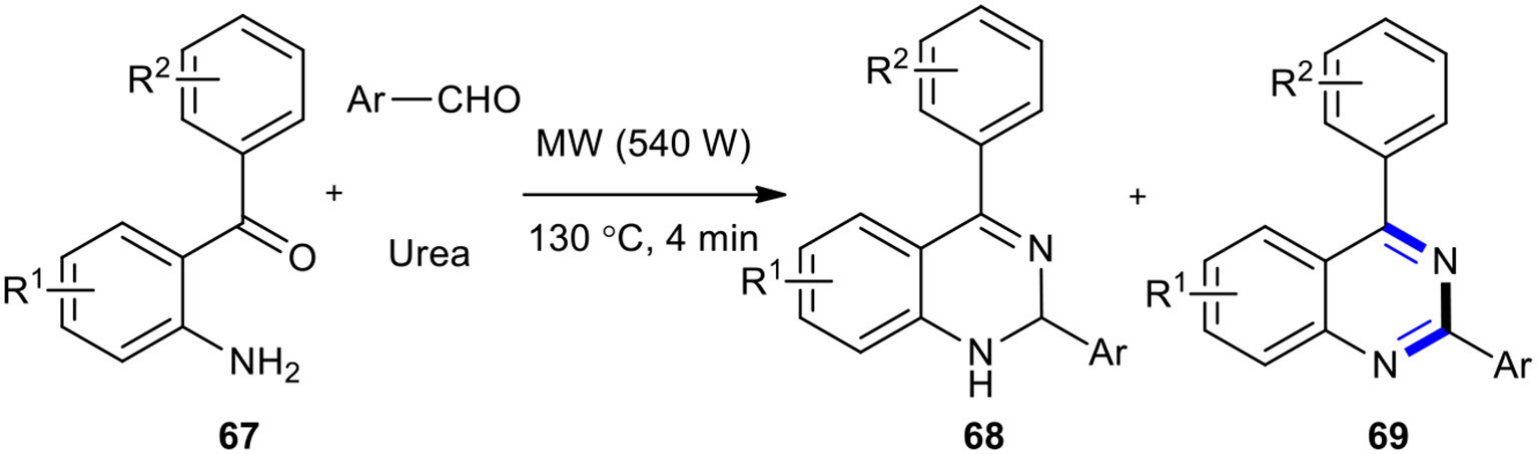
MW–assisted MCR of **67**, aldehydes, and urea to afford **69**.

When 2-aminobenzophenones **67** were reacted with aldehydes and urea as an environmentally benign source of ammonia through one-pot three-component reaction under MW conditions (540 W and 130°C), a library of 2,4-disubstituted-1,2-dihydroquinazolines **68** as a major product with a small amount of quinazolines **69** as a minor product were obtained (Scheme 25) (Sarma and Prajapati, 2011). Their methodology also employed ammonium acetate as a good source of ammonia in place of urea. The salient features of this method were that it was rapid, simple, and clean, and had no need for a solvent or catalyst.

## Synthesis of Quinazolinone Derivatives

### Radziszewski's Reaction Using UHP

**Scheme 26:**
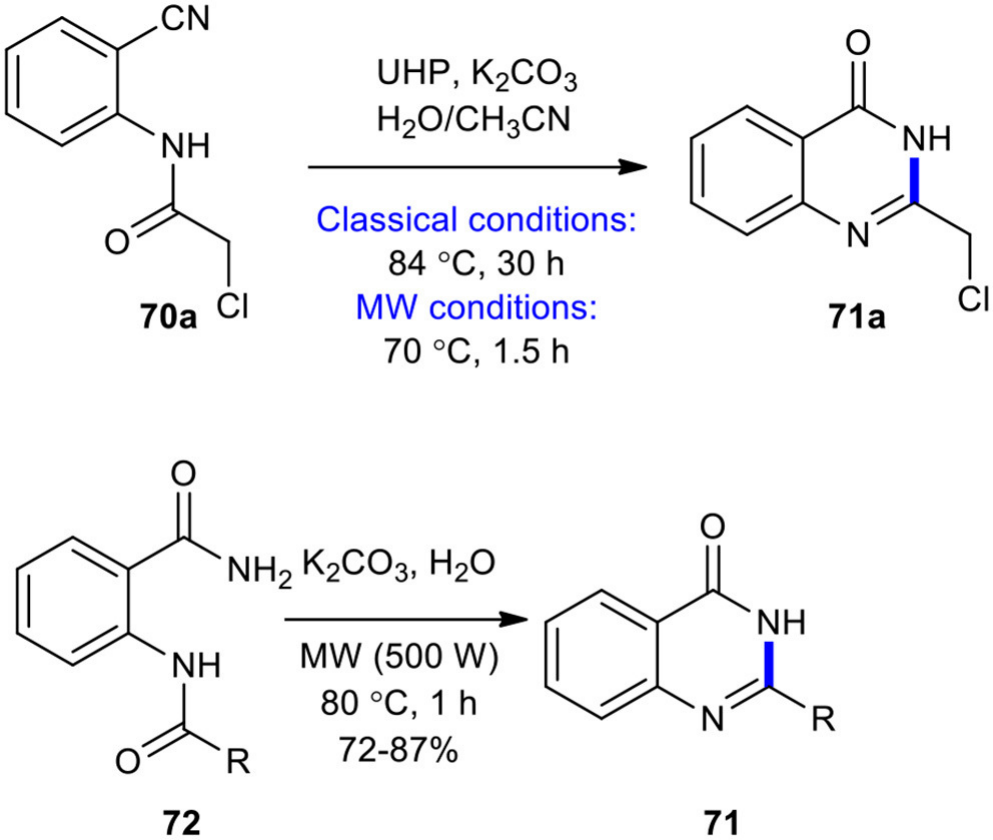
Radziszewski's reaction using UHP under conventional and MW conditions to afford **71a**.

The Radziszewski reaction is an organic reaction which involves the oxidation of nitriles using alkaline hydroperoxide (Radziszewski, 1885). Performing the reaction on *o*-amidobenzonitriles may result in the formation of quinazolin-4-(3*H*)-ones (Bogert and Hand, 1902). The Radziszewski reaction as a useful and efficient strategy has been employed in the synthesis of quinazolinones under MW conditions. For example, MWI was used in the cyclization of 2-chloro-*N*-(2-cyanophenyl)acetamide (**70a**) and 2-amidobenzamide derivatives **72** to quinazolinones (Kabri et al., 2009). It is noteworthy that compounds **70** and **72** were obtained as a solid since they could be directly converted into the target molecules. Compound **70a** was subjected to Radziszewski's reaction in the presence of urea hydrogen peroxide (UHP) as a safe, mild, and non-hazardous oxidizing agent in the mixture of acetone/H_2_O (1:1, v/v) to afford 2-chloromethylquinazolin-4(3*H*)-one **71a**. The reaction was performed under MW and classical conditions. In classical conditions, the product **71a** was formed in a 55% yield after 30 h reaction time at 84**°**C, whereas, under MW conditions (500 W, 70***?***C, 1.5 h), the same product was obtained in a 78% yield. Cyclization of derivatives **72** to quinazolin-4(3*H*)-ones **71** was accomplished when K_2_CO_3_ in water was used under MWI at 80?C for 1 h at a power of 500 W, obtaining the expected compounds in good yields (Scheme 26).

### Double Cyclization

**Scheme 27:**
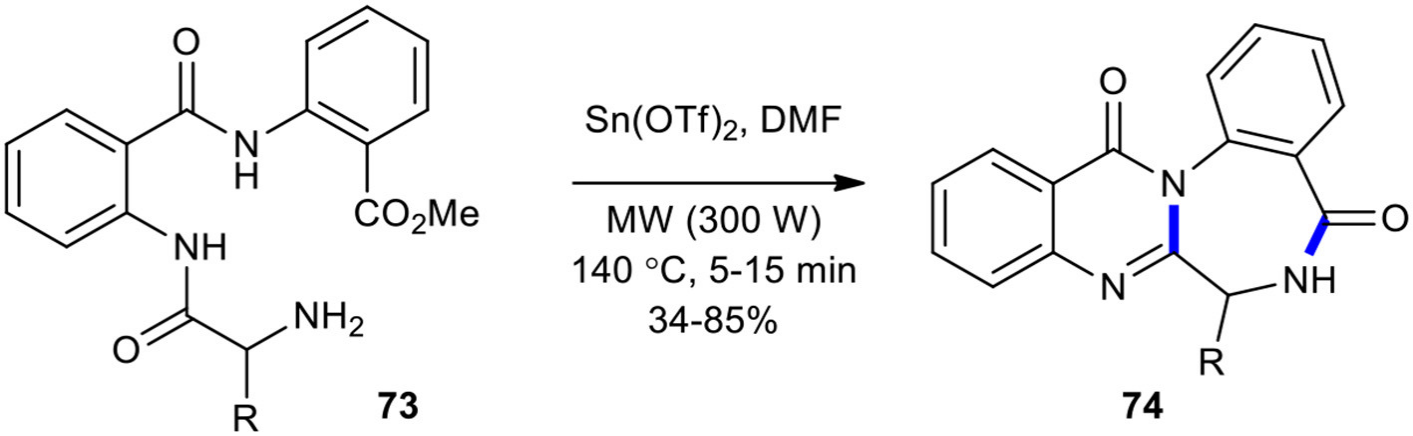
MW–assisted direct double cyclization of **73** to afford **74**.

A double cyclization reaction was employed in the synthesis of natural products containing quinazolinone under MW conditions. For example, the direct double cyclization of tripeptides **73** using Sn(OTf)_2_ in DMF and MWI at 140°C to access the structurally more challenging members, quinazolinobenzodiazepines **74**, including sclerotigenin, asperlicin C, and circumdatin F natural products and their analogous compounds, in 5–15 min was accomplished within a week to good yields (34–85%) (Scheme 27) (Tseng et al., 2009). Tin(II) triflate in this dehydrative cyclization reaction could serve as an effective Lewis acid.

### Intramolecular Azido-Reductive Cyclization

**Scheme 28:**
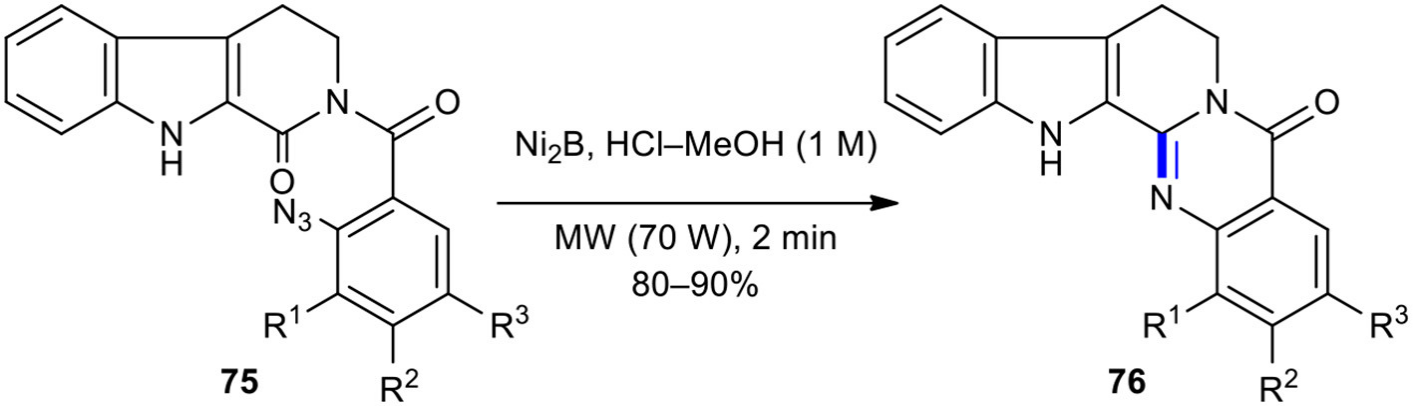
MW–assisted intramolecular azido-reductive cyclization of **75** to afford **76**.

Intramolecular azido-reductive cyclization strategy has previously been employed in the synthesis of natural products under MW conditions. For example, Kamal et al. developed a simple, mild, and efficient synthetic route to synthesize rutaecarpine, euxylophoricines A and C, and several analogs by using an intramolecular azido-reductive cyclization assisted by MWI of the corresponding substituted azido cyclic amides **75** as precursors (Kamal et al., 2011). The reduction of azido functionality in 2,3,4,9-tetrahydro-β-carbolin-1-one derivatives **75** with triphenylphosphine (PPh_3_) or nickel boride (Ni_2_B) as a reducing reagent in HCl–MeOH using a CEM Discovery MW reactor furnished the expected quinazolinones **76** in excellent yields (80–90%) (Scheme 28).

### Niementowski Quinazoline Synthesis

**Scheme 29:**
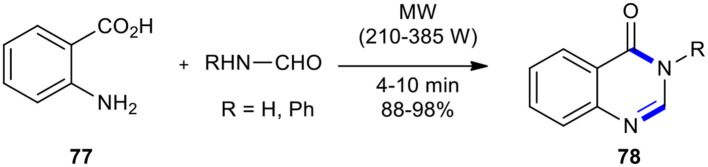
MW–assisted Niementowski reaction to afford **78**.

The Niementowski quinazoline synthesis involves the reaction of anthranilic acids with amides to construct 4-oxo-3,4-dihydroquinazolines (3*H*-quinazolin-4-ones). It is the most commonly used synthetic method to form the 3*H*-quinazolin-4-one ring. This synthetic route generally involves lengthy and tedious conditions as well as high temperatures. MWI as a powerful technique is capable of improving and reducing reaction times and increasing the yield of the reaction more than the purely thermal heating source. The first highly accelerated Niementowski reaction of anthranilic acid (**77**) with formamide (or formanilide) using a house-hold unmodified microwave oven under solvent-free conditions was reported by Khajavi et al., leading to the high purity of quinazolinone derivatives **78** (Scheme 29) (Khajavi et al., 1998). The salient features of this method included operational simplicity and a simple work-up procedure.

**Scheme 30:**
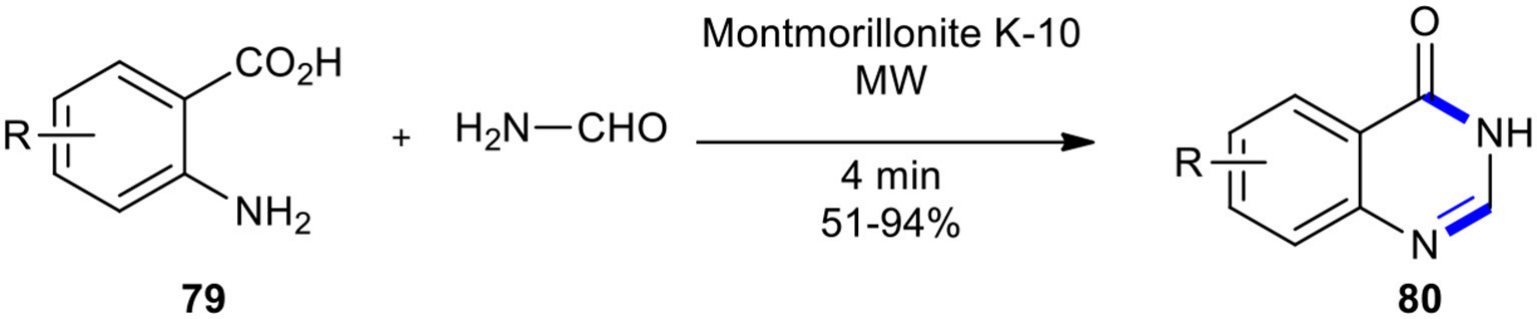
MW–assisted Niementowski reaction of **79** and formamide using MK-10 to afford **80**.

As a general, efficient, and useful method, the combination of supported reagents and MWI was used in solvent-free conditions to prepare the requested quinazolines **80** through Niementowski quinazoline synthesis (Balalaie et al., 2001). The reaction of anthranilic acids **79** with formamide under MW conditions for 4 min was achieved using acidic alumina, silica gel, and montmorillonite K-10 (MK-10). Among acidic solid-supported reagents, montmorillonite K-10 gave the best yield (Scheme 30). Solvent-free conditions, good yields, short reaction times, and a simple set-up and work-up procedure are advantages of this method.

**Scheme 31:**
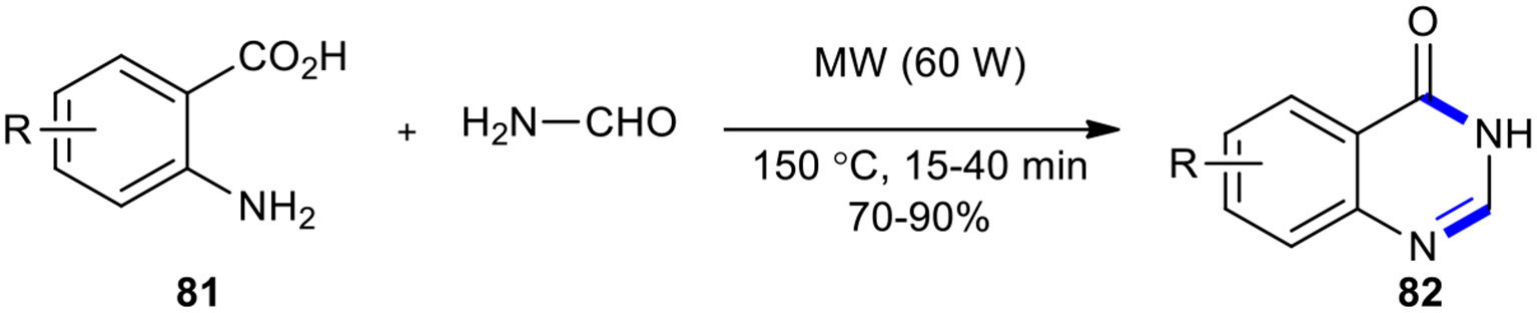
MW–assisted Niementowski reaction using an excess of formamide to afford **82**.

The Niementowski reaction under MWI (60 W) was employed for the synthesis of 3*H*-quinazolin-4-one core **82** using an excess of formamide (5 equiv) as a fusion accelerator in a fixed temperature (150°C) (Scheme 31) (Alexandre et al., 2002). The expected products **82** were obtained in very good yields without any by-products.

**Scheme 32:**
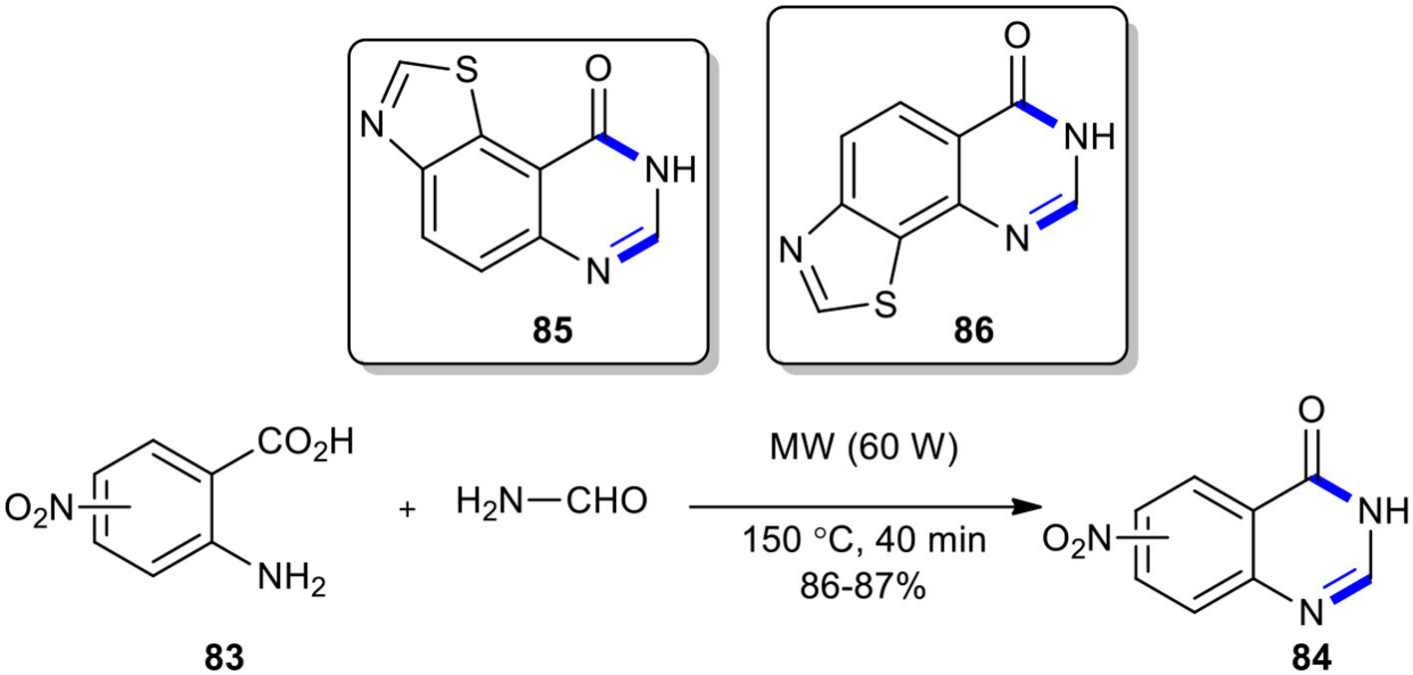
The synthesis of **84** through MW–assisted Niementowski reaction toward **85** and **86**.

Alexandre et al. described an original synthetic route to the rare 8*H*-thiazolo[5,4-*f* ]quinazolin-9-one (**85**) and the novel 7*H*-thiazolo[4,5-*h*]quinazolin-6-one (**86**), starting with the formation of 3*H*-nitroquinazolin-4-ones **84** (Alexandre et al., 2003a). The MW–assisted Niementowski condensation using formamide under MW conditions (60 W, 150°C) for 40 min annulated rapidly the quinazolin-4-one rings **84** which, in several steps, were converted into products **85** and **86**, with different functional group in different positions (Scheme 32).

**Scheme 33:**
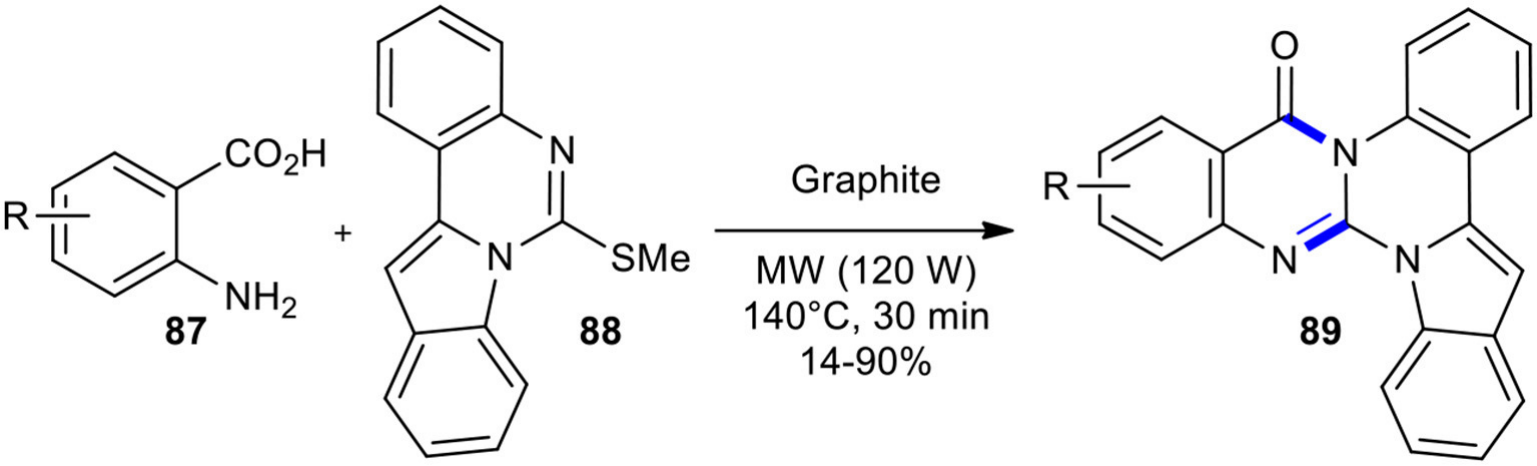
MW–assisted synthesis of **89** from **87** and **88**.

Domon et al. prepared new triazabenzo[*a*]indeno[1,2-*c*]anthracen-5-ones and triazabenzo[*a*]indeno-[1,2-*c*]naphtacen-5-one **89** using MWI in dry media (Domon et al., 2001). Cyclization of anthranilic acid derivatives **87** using indoloquinazoline derivative **88** was performed with an excess of anthranilic acid, adsorbed on graphite, using MWI (120 W) at 140°C for 30 min (Scheme 33). Interestingly, they realized that the direct condensation of anthranilic acids with thioamides may be very difficult or unsuccessful; hence, they converted the mercapto group of thioquinazolines into a better leaving group. Their method gave products **89** in various yields without any by-products. Although MWI shortened the reaction time and provided a cleaner reaction compared to the same reaction in purely thermal procedures, it should have been performed at a higher temperature (140°C) due to graphite/MW interaction.

**Scheme 34:**
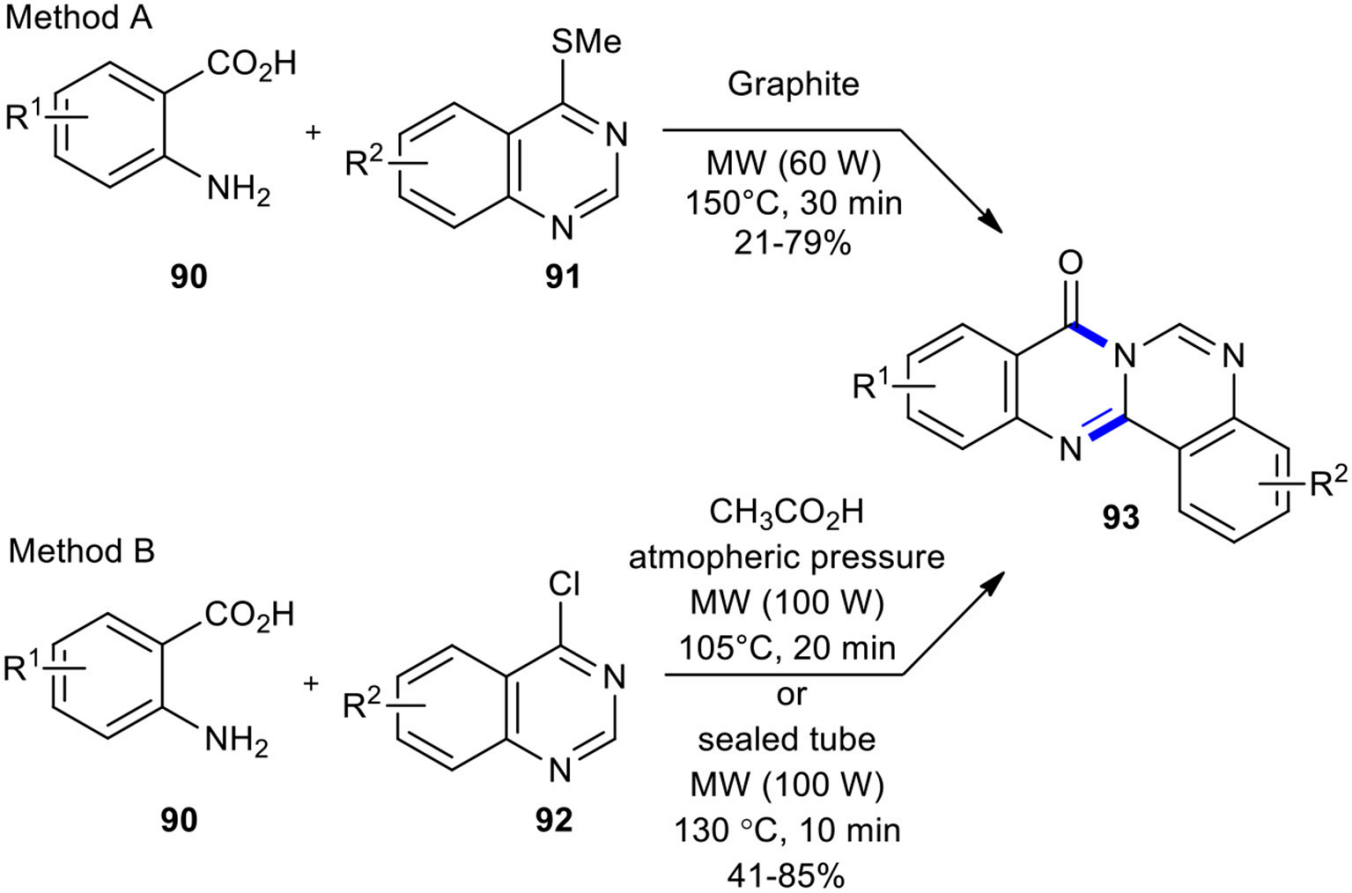
MW–assisted synthesis of **93** from **91** or **93**.

To access 8*H*-quinazolino[4,3-*b*]quinazolin-8-ones **93**, Alexandre et al. introduced two different leaving groups on the quinazoline ring (Scheme 34) (Alexandre et al., 2003b). In method A, 4-(thiomethyl)quinazolines **91** was condensed with anthranilic acids **90** on graphite under the optimized reaction conditions (MW, 60 W, 150°C, 30 min). In method B, the use of chlorine instead of thiomethyl group offered more straightforward access to target molecules **93** and afforded better yields than method A. It is noteworthy that method B employed acetic acid as a solvent while method A occurred in solvent-free conditions.

**Scheme 35:**
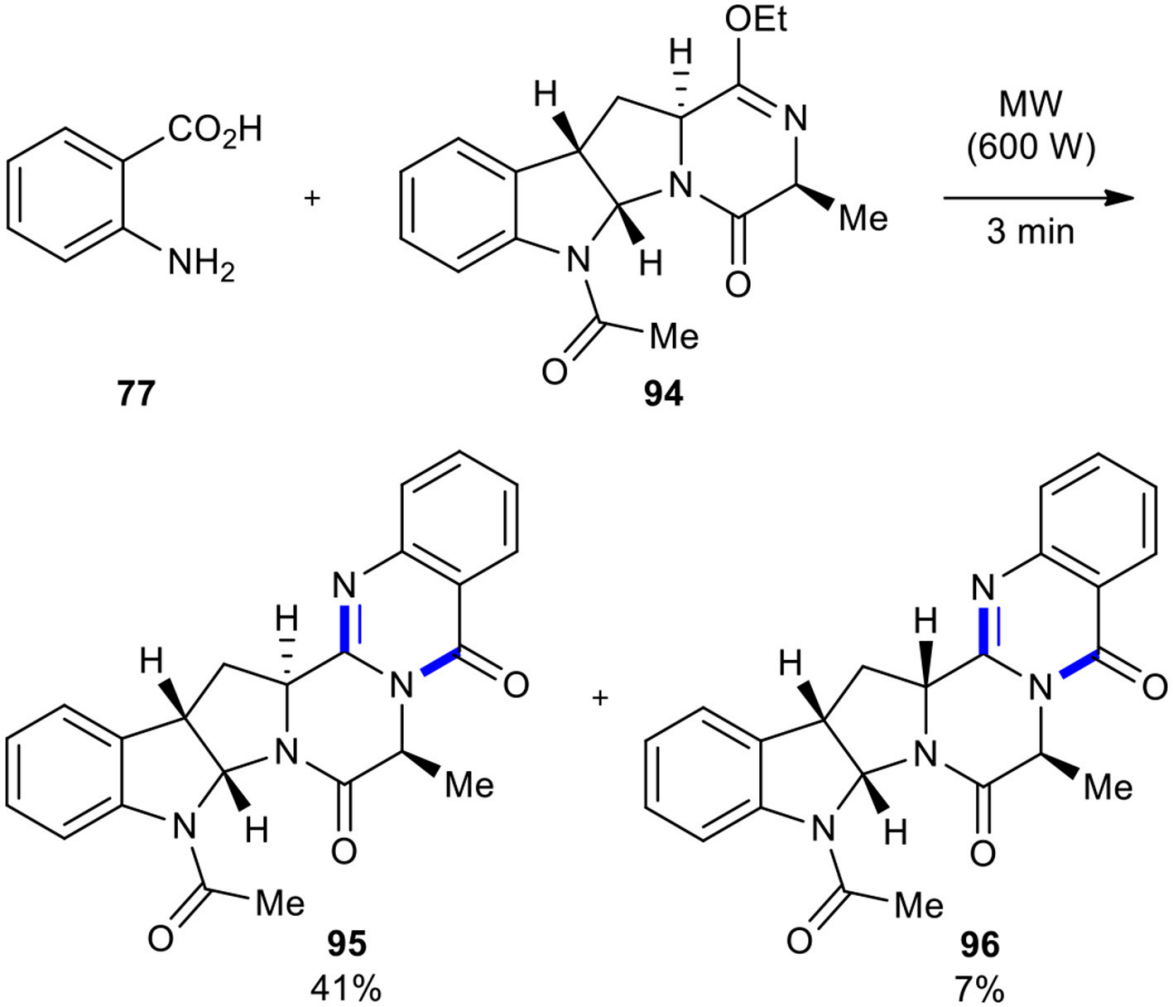
MW–assisted modified Niementowski condensation of **94** to afford **95** and **96**.

The reaction of anthranilic acid with lactim ethers is one of the methods to synthesize the quinazoline scaffold. This reaction was relatively neglected in the past, due to its low yields and the possibility of epimerization of stereocenters adjacent to carbonyl groups (Rajappa and Advani, 1973, 1974; Caballero et al., 1998) when performed under thermal and solvent-free conditions. Cledera et al. improved the earlier protocol by utilizing MWI in the cyclocondensation of anthranilic acid with lactim ethers, preparing pyrazino[2,1-*b*]quinazoline-3,6-diones (Cledera et al., 2004). When the tetracyclic ardeemin fragment **94** was reacted with anthranilic acid (**77**) under MW conditions (600 W) for 3 min, a 6:1 mixture of the diastereoisomeric *de*-prenylardeemins **95** and **96** in 48% overall yield was obtained (Scheme 35). The results showed that the optimized reaction conditions not only improved yield and shortened the reaction time but also improved stereochemical integrity compared to the conventional conditions.

**Scheme 36:**
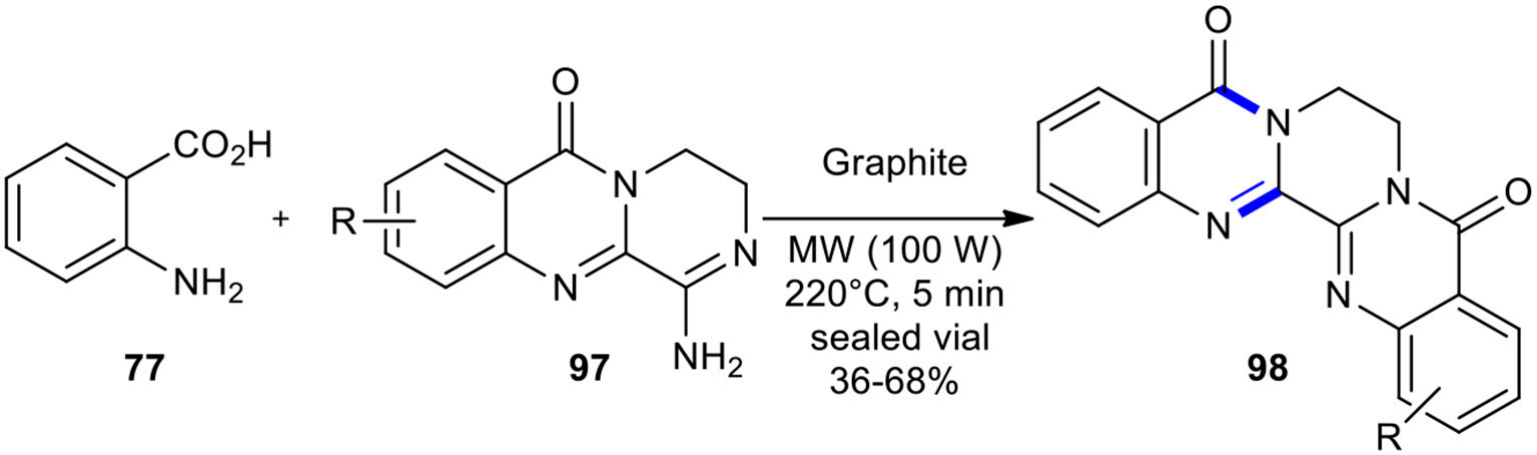
MW–assisted modified Niementowski condensation of **97** to afford **98**.

Novel tetraaza-pentaphene-5,8-dione derivatives **98** could be synthesized from anthranilic acid (**77**) and the 2,3-condensed (3*H*)-quinazolin-4-ones **97** *via* a MW–assisted modified Niementowski condensation under pressure at 220°C (Scheme 36) (de Fatima Pereira et al., 2005).

**Scheme 37:**
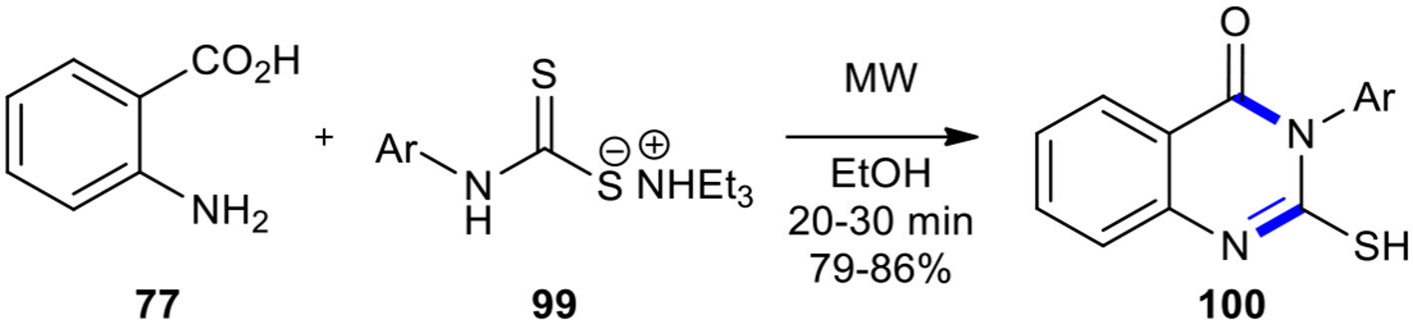
MW–assisted modified Niementowski condensation of **99** to afford **100**.

An efficient method for forming a series of 3-aryl 2-thioderivatives of quinazolinone **100** through the MW-promoted cyclocondensation of anthranilic acid (**77**) with thio carbamate salt of variously substituted anilines **99** in either solvent (ethanol) or solvent-free conditions was achieved (Scheme 37) (Patil et al., 2012). The result showed that during the operation of MWI, better yields were obtained in the presence of a solvent or solventless system although, under solvent-free conditions, the reaction time was shortened from 20–30 to 2–3 min. As expected, the MWI, compared to conventional heating, significantly reduced the reaction time and improved the reaction yield.

#### Niementowski Modification of Friedlander Synthesis

**Scheme 38:**
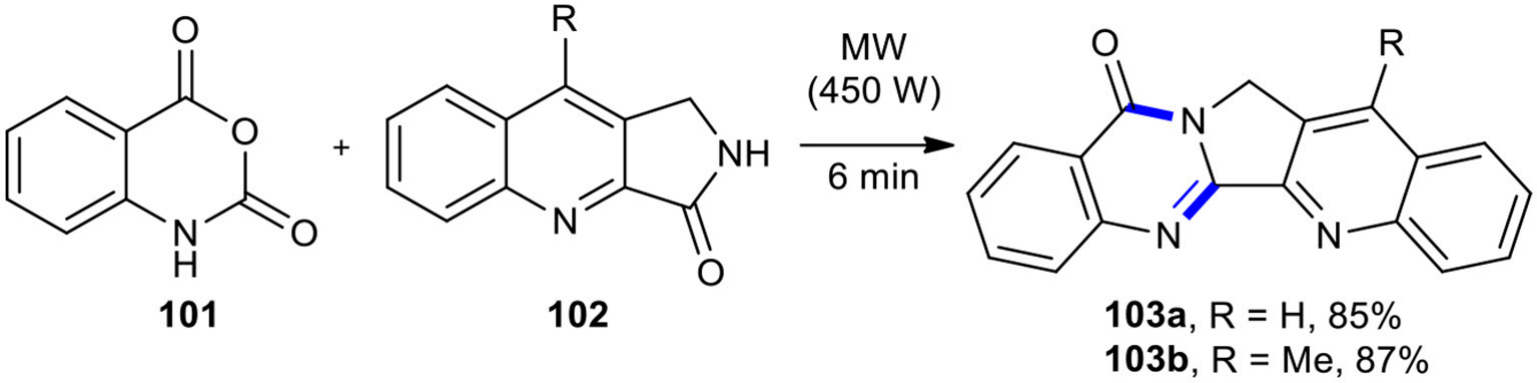
Niementowski modification of Friedlander synthesis of **102**.

The use of isatoic anhydride in place of anthranilic acid in the Niementowski reaction results in the Niementowski modification of Friedlander synthesis. The Niementowski modification of Friedlander synthesis has been employed in the synthesis quinazolinone scaffold. Yadav and Reddy reported the total synthesis of the cytotoxic alkaloid luotonin A (**103a**) for the first time in high yields based on the reaction of isatoic anhydride (**101**) with 3-oxo-1*H*-pyrrolo[3,4-*b*]quinoline (**102**) under MWI and solvent-free conditions (Yadav and Reddy, 2002). Using the present reaction conditions, methyl-substituted luotonin A (**103b**) was produced in good yield (87%) (Scheme 38). This process was new, efficient, simple, clean, rapid, and higher yielding than the reported strategies for the preparation of luotonin A. This approach used inexpensive reagents in this synthesis.

**Scheme 39:**
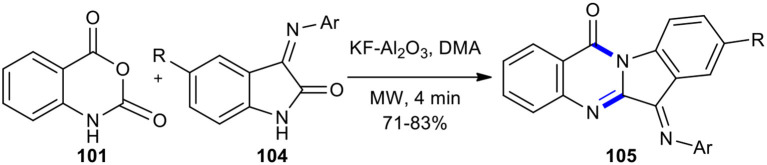
MW–assisted Niementowski modification of the Friedlander synthesis of **104**.

Azizian et al. previously prepared quinazolinone derivatives from isatin-3-imine and isatoic anhydride using MWI (Azizian et al., 2004a). The MW–assisted condensation of isatin-3-imines **104** with isatoic anhydride (**101**) using KF on alumina as a reusable catalyst in DMA in 4 min resulted in the formation of the new 6-arylimino-6*H*-indolo[2,1-*b*]quinazolin-12-ones **105** (Scheme 39). The reaction proceeded through ring-opening and cyclization.

**Scheme 40:**
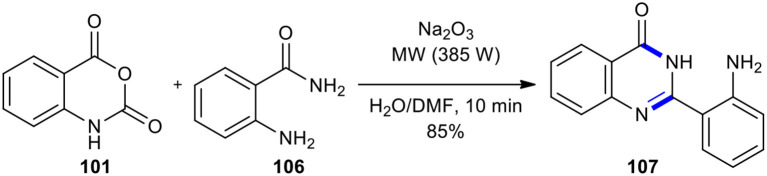
MW–assisted Niementowski modification of the Friedlander synthesis of **106**.

Later, the same group used an MW–assisted Niementowski modification of the Friedlander synthesis (Pater, 1971) to prepare 2-(*o*-aminophenyl)-4(3*H*)-quinazolinone (**107**) (Azizian et al., 2004b). When isatoic anhydride (**107**) was reacted with anthranilamide (**106**), the nucleophilic attack of the *o*-amino group of the anthranilamide opened the isatoic anhydride ring which upon reaction with Na_2_CO_3_ (*eq*.) gave 2-(*o-*aminophenyl)-4(3*H*)-quinazolinone (**107**) (Scheme 40).

### Metal-Catalyzed Cyclization of Benzoic Acids

Catalysis is the process of enhancing the rate of a chemical reaction by adding a substance known as a catalyst (McNaught and Wilkinson, 1997; Masel, 2001). Generally, metal catalysts are recognized as the most important factors in increasing catalyzed organic transformations. Particularly, transition metal-catalyzed reactions are fundamental for the convenient and effective synthesis of a wide range of organic compounds (Beller et al., 1998; Bates, 2012). The area of transition metal catalysis indisputably had an immense impact on basic research in academia and chemical industries, thus in turn having an effect on modern society and our daily life. That is well-recognized through several awarded Nobel Prizes during the twentieth century, stretching from Nobel Prize (2020) for the synthesis of ammonia to Richard Suzuki, Heck, and Negishi, who shared the 2010 the prize for their outstanding endeavors and achievements in transition metal-catalyzed cross-coupling reactions (Johansson Seechurn et al., 2012). Although the aforementioned reactions are considered as the most important transition-metal catalyses, a plethora of vital reactions can be effectively catalyzed by various transition metals including reductive elimination, oxidative addition, and transmetallation. Transition metal catalysis has also found extensive applications in the convenient and efficient synthesis of a wide variety of heterocyclic systems (Ma et al., 2017; Ramanathan and Liu, 2017; Tiwari and Bhanage, 2017; Chatterjee et al., 2018; Saikia et al., 2018; Chen et al., 2020; Debabrata et al., 2020; Ghosh et al., 2020; Janardhanan et al., 2020; Jiang et al., 2020; Kanwal et al., 2020; Kojima and Matsunaga, 2020; Li and Zhang, 2020; Nagata and Obora, 2020; Neto and Zeni, 2020a,b; Pal et al., 2020; Ratmanova et al., 2020; Sahiba and Agarwal, 2020; Sonawane et al., 2020; Xuan et al., 2020). However, the literature survey revealed only an example of zinc (Shi et al., 2004), a limited number of iron-catalyzed (Melvin et al., 1992; Valderrama et al., 1999; Kanth et al., 2006; Yin et al., 2012; Gopalaiah et al., 2017, 2019; Raut and Bhanage, 2017; Eidi et al., 2018), and relatively more copper-catalyzed reactions, leading to the synthesis of quinazoline and quinazolinone derivatives (Melvin et al., 1992; Chatterjee et al., 2018; Potuganti et al., 2018; Liang et al., 2019; Rodrigues et al., 2019; Donthiboina et al., 2020). The number of zinc, iron, and copper-catalyzed synthesis of quinazoline and quinazolinone derivatives were decreased when the literature survey was narrowed down to the choice of those reactions, performed specifically under MWI as an unconventional and environmentally benign source of energy, in accordance with the title of this review.

**Scheme 41:**
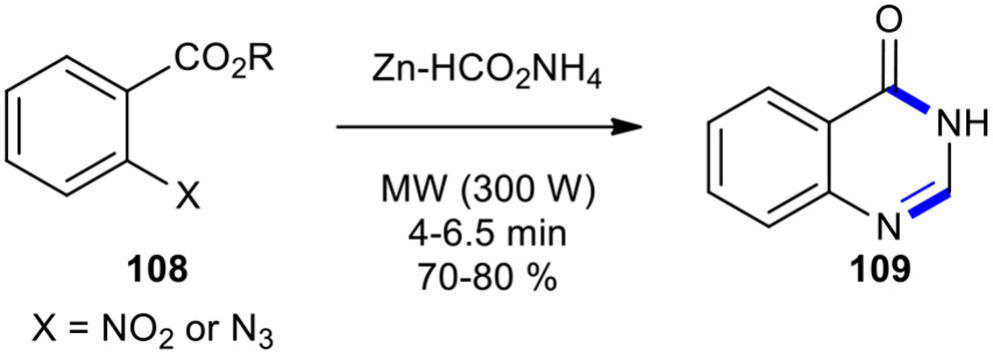
One-pot MW–assisted Zn-catalyzed reduction and cyclocondensation of nitro and azido arenes **108** to produce **109**.

In this regard, the 2-nitro- and 2-azido substituted benzoic acid derivatives have been employed as substrates for the synthesis of quinazolinones. A simple, efficient, and mild method for the synthesis of (3*H*)-quinazolinone (**109**) under solvent-free conditions based on the MW-mediated reduction of nitro and azido arenes to *N*-arylformamides using Zn and ammonium formate (Zn–HCO_2_NH_4_) was developed by Kamal et al. (2004). To produce (3*H*)-quinazolinone **109**, the 2-substituted azido- or nitrobenzoic acids **108** were transformed into their corresponding *N*-arylformamides **109** by employing Zn–HCO_2_NH_4_ under MWI at 300 W (Scheme 41). In this reaction, Zn acted as an efficient and inexpensive catalyst, while HCO_2_NH_4_ was decomposed to formamide, which in the following was condensed with the anthranilic acid. It is important to note that the reaction in the absence of MWI afforded amines instead of arylformamides.

As described above, apart from palladium and other transition metals, copper and iron species are catalysts of choice in the synthetic community. In general, they are advantageous, expedient, cost-effective, and relatively less toxic. Thus, iron (Correa et al., 2008; Li et al., 2013; Ghorai et al., 2017) and copper-catalyzed (Monnier and Taillefer, 2013) organic transformations have recently received enormous attention from synthetic organic research groups; they especially are the catalysts of choice for organic transformations requiring, C–N bond formation. In this regard, in recent years, applications of copper and iron-catalyzed transformations have overgrown and still are developing. They have been used in the synthesis of a wide variety of heterocyclic systems, especially *N*-heterocycles. In this regard, they have been used as effective catalysts in the synthesis of quinazoline and quinazolinone derivatives under different reaction conditions, involving conventional heating or green sources of energy such as ultrasound or MWI.

The reaction of 2-halobenzoic acids and ammonia as a source of nitrogen catalyzed either iron or copper in the presence of a base such as Cs_2_CO_3_ and NaOH leads to the construction of quinazolinones (Zhang et al., 2009; Ke et al., 2018; Radhakrishnan et al., 2018). However, in these reactions, stoichiometric amounts of bases were often necessary, and in some cases using ligands was also essential (Ley and Thomas, 2003; Corbet and Mignani, 2006). Since the scope of this review is limited to underlining the synthesis of quinazolinone and quinazolinone derivatives, successfully performed under MWI as a green source of energy (Zhou et al., 2008; Liu et al., 2009; Zhang et al., 2009; Xu and Fu, 2011, 2012; Xu et al., 2011; Sreenivas et al., 2012), in the following we try to describe the synthesis of the above-mentioned heterocycles being successfully conducted in the presence of either Fe or Cu species under MWI.

**Scheme 42:**
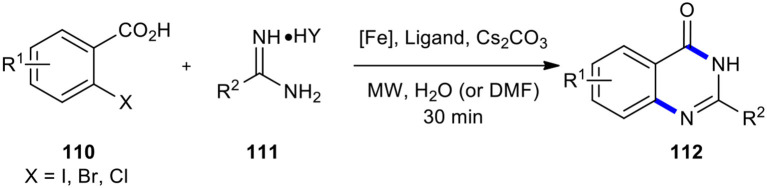
MW–assisted iron-catalyzed cyclization of **110** with **111** to afford **112**.

The 2-halobenzoic acids have been employed as substrates for transition metal-mediated cross-coupling and cyclization with urea derivatives to synthesize the 2-substituted quinazolinones in a single-pot operation. For example, Zhang et al. performed the reaction of substituted 2-halobenzoic acids **110** with amidines **111** in the presence of a Fe species, ligand, and base in a suitable solvent using MW heating to obtain quinazolinone derivatives **112** (Scheme 42) (Zhang et al., 2009). Worth noting is that the above reaction was conducted diversely with or without ligand in water or DMF, which afforded the respective products in moderate to high yields. As expected, 2-iodobenzoic acids were more reactive than 2-bromobenzoic acids and 2-chlorobenzoic acids. The features of this method included being green, rapid, highly efficient, versatile, inexpensive, and environmentally friendly, especially when water as a solvent is used.

**Scheme 43:**
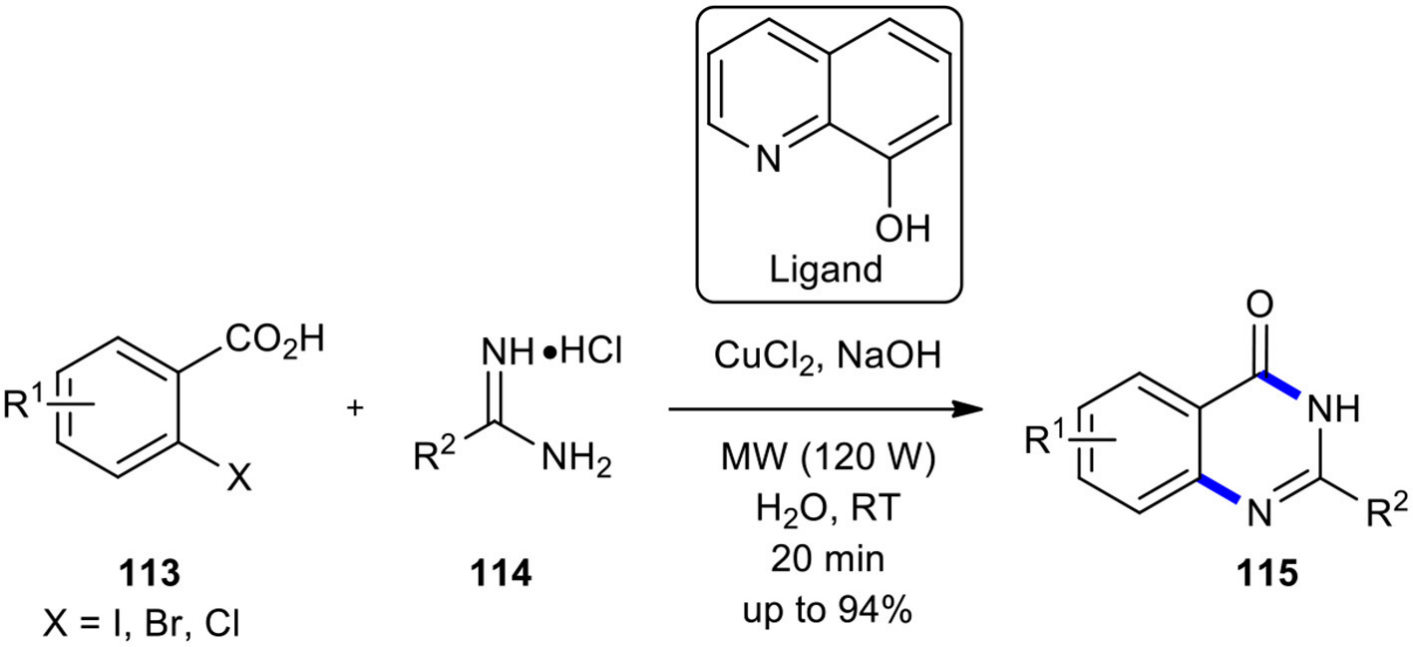
MW–assisted copper-catalyzed cyclization of **113** with **114** to afford **115**.

Copper continues to be one of the most employed and popular transition metal catalysts in synthetic organic chemistry. Furthermore, to its economic and environmental benefit over other transition metal catalysts, it is also abundant. Thus, nowadays, copper catalysis has attracted enormous attention from synthetic organic chemists. Several review articles collected and described the recent progress made in pleasing and exceptional copper catalysis in all areas of synthetic transformations (Monnier and Taillefer, 2013; Guo et al., 2015; Thapa et al., 2015; Tandon, 2019; Ghiazza and Tlili, 2020). Copper-based catalysts are also extensively employed in different chemical industries (Punniyamurthy and Rout, 2008). In this line, Ke et al. used copper as a catalyst instead of iron in the reaction of 2-halobenzoic acids with amidines (Ke et al., 2018). The synthesis of 37 examples of quinazolinones **115** was achieved in good to excellent yields (up to 94%) in 20 min when the substituted 2-halobenzoic acids **113** were coupled with amidines **114** in the presence of CuCl_2_, ligand, and NaOH in water under MWI at 120 W at room temperature (Scheme 43). The remarkable features of this strategy were using an inexpensive and commercially available and environmentally benign copper catalyst, activation at low temperatures, and there being no need for an inert atmosphere. Worth noting is that the relative reactivity of 2-halobenzoic acids was similar to that of Zhang's method, using iron species as catalyst (Zhang et al., 2009).

**Scheme 44:**
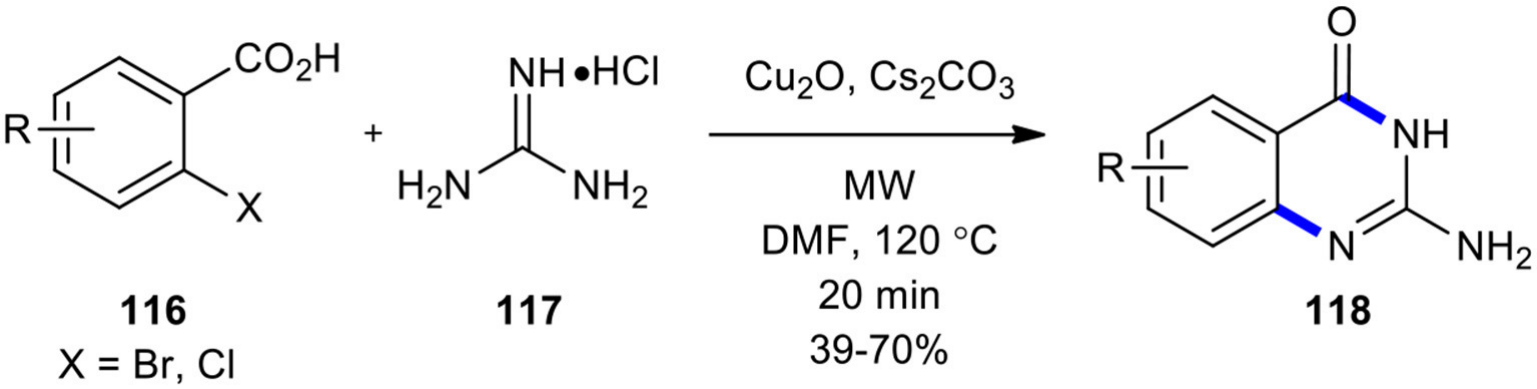
MW–assisted copper-catalyzed cyclization of **116** with **117** to produce **118**.

In the following, Cu-catalyzed one-pot coupling and cyclization leading to the construction of *N*-heterocycles was studied (Radhakrishnan et al., 2018). The reaction of 2-halobenzoic acids **116** with guanidine hydrochloride (**117**) using MWI resulted in the formation of 2-aminoquinazoline analogs **118** (Scheme 44). The target products **118** were obtained in one-pot with high yield without the need of any additional ligand. Cu_2_O and Cs_2_CO_3_ proved to the best catalyst and base, respectively, for the MW–assisted Cu(I)-catalyzed reaction to form C-N bond. The advantages of C-N bond formation strategy are the same as described for Ke's method (Ke et al., 2018); there was also no need for any ligand.

### Cyclocondensation Using Different Methods

MWI has been employed to promote cyclocondensation of various nitrogen-containing precursors with ortho-esters, aldehydes, carboxylic acids, TEAF, benzyl alcohols, amines, thioamides, isothiocyanates, and potassium isopropyldithiocarbonate, as described below.

#### Ortho-esters

**Scheme 45:**
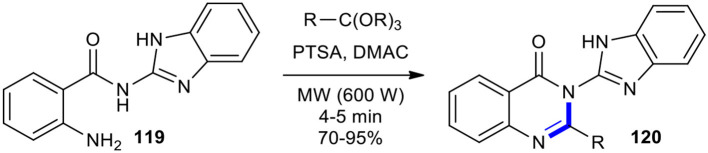
MW–assisted synthesis of **120** from **119** and ortho-esters using PTSA.

Ortho-esters have been condensed with 2-carbonyl substituted aniline derivatives under MW conditions to create variously substituted quinazolinones and their heterocycle-appended hybrids. Hazarkhani and Karimi synthesized a variety of new 3-(2-benzimidazolyl)-2-alkyl-4-(3*H*)-quinazolinones **120** in good to high yields in a facile and rapid manner through the reaction of 2-amino-*N*-(1-*H*-benzimidazol-2-yl)benzamide (**119**) with a set of ortho-esters under MWI (600 W) in the presence of *p*-toluenesulphonic acid (*p*-TsOH or PTSA) in DMAC (Scheme 45) (Hazarkhani and Karimi, 2003).

Numerous methods for the construction of the quinazolinone skeleton based on the cyclocondensation of 2-aminobenzamides with orto-esters using MWI have been reported in the literature. A variety of conditions in the absence of a solvent to the quinazolinone skeleton have been used. These make use of solid catalyst systems such as (SiO_2_/H_2_SO_4_) (Montazeri and Rad-Moghadam, 2004), montmorillonite K-10 (Dabiri et al., 2004b), AlCl_3_/ZnCl_2_-SiO_2_ (Dabiri et al., 2005), and HY-zeolite (Bakavoli et al., 2007; Montazeri et al., 2012a), as well as organocatalysts, including pentafluorophenylammonium triflate (PFPAT) (Montazeri et al., 2012b).

#### Aldehydes

**Scheme 46:**
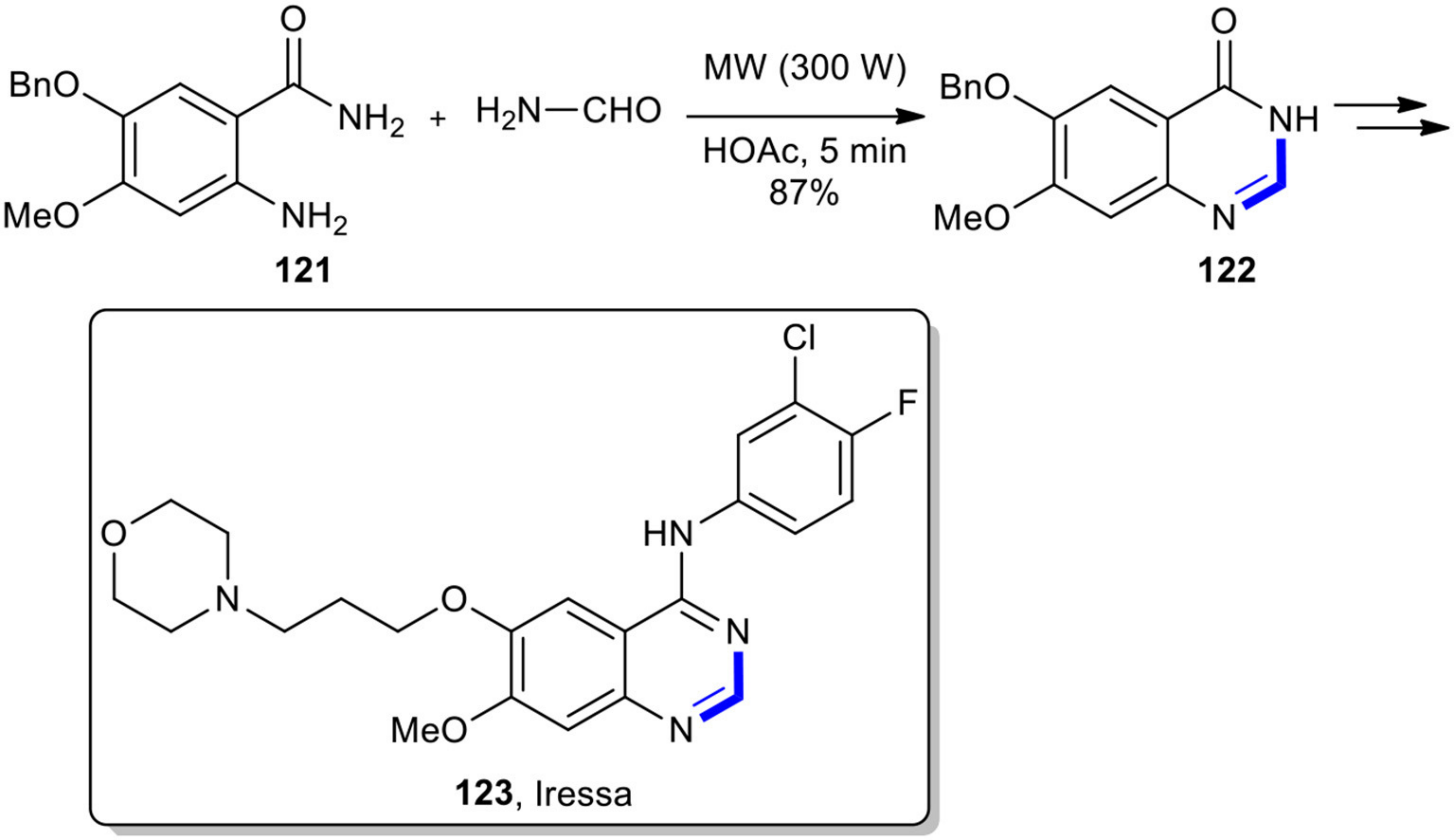
MW–assisted synthesis of **122** from **121** and formamide.

Anthraniamide derivatives have also been condensed with various aldehydes under MW conditions to afford quinazolinones. The highly accelerated Niementowski synthesis of quinazolin-4(3*H*)-one using MW heating protocol as a key step was successfully applied for the construction of an important and key anticancer drug, Iressa (**123**) (Li et al., 2007). The 6,7-disubstituted quinazolin-4(3*H*)-one **122** was prepared in an 87% isolated yield from a 5-benzyloxy-4-methoxy-2-aminobenzamide (**121**) and formamide under the acidic catalytic amount of acetic acid by MWI at 300 W for 5 min, termed as the Niementowski synthesis. After several steps, the key intermediate **122** was converted into Iressa (**123**) as a pale yellow solid (Scheme 46). This method could improve the reaction conditions from 5 h of heating at 190°C to 5 min using MWI.

**Scheme 47:**
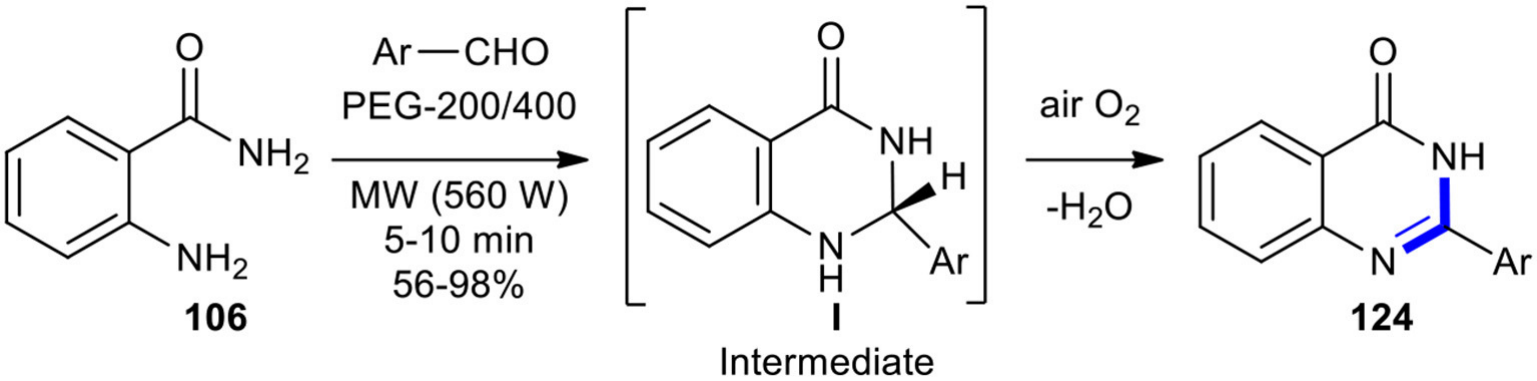
MW–assisted synthesis of **124** using PTSA in PEG as a solvent.

The condensation of anthranilamide (**106**) with different aromatic aldehydes in the presence of PTSA in polyethylene glycol (PEG-200/400) as a green solvent without the use of an oxidant under MW conditions (560 W in a domestic microwave oven) for 5–10 min afforded the corresponding 2-aryl- or 2-hetaryl-4(3*H*)-quinazolinones **124** in good to excellent yields with high purity (Scheme 47) (Deligeorgiev et al., 2010). This method provided several benefits, including novel environmentally friendly conditions, having a simple work-up procedure, carrying out the reactions in very short times, and isolating products only by filtration.

**Scheme 48:**
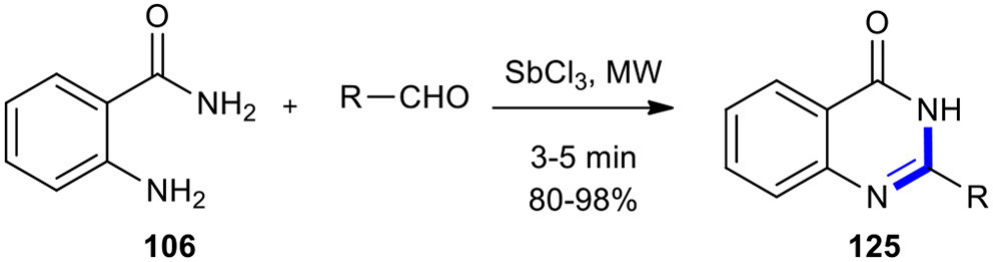
MW–assisted synthesis of **125** using SbCl_3_.

Recently, Kang et al. reported the MW–assisted synthesis of quinazolin-4(3*H*)-one derivatives **125** within several minutes under solvent-free and mild conditions (Kang et al., 2018). The products could be obtained in good to excellent yields when anthranilamide (**106**) was condensed with various aldehydes in the presence of a catalytic amount of commercially available antimony (III) trichloride (SbCl_3_) (Scheme 48).

#### Carboxylic Acids

**Scheme 49:**
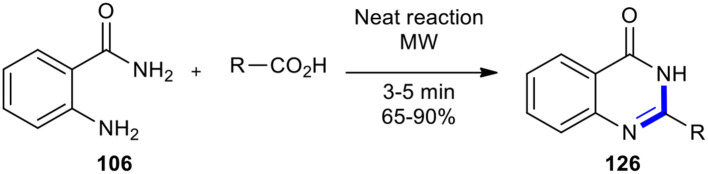
MW–assisted synthesis of **126** from **106** and carboxylic acids.

Carboxylic acids have also been employed as reactants with 2-substituted anilines to afford quinazolino-4-one derivatives. MW-assisted cyclocondensation of 2-aminobenzamide (**106**) with carboxylic acids was achieved in 3-5 min under solvent-free conditions to afford a series of 2-substituted quinazolin-4(3*H*)-ones **126** as represented in Scheme 49 (Rahimizadeh et al., 2004). This transformation was 40-80 times faster than under conventional heating methods and obtained products in higher yields.

**Scheme 50:**
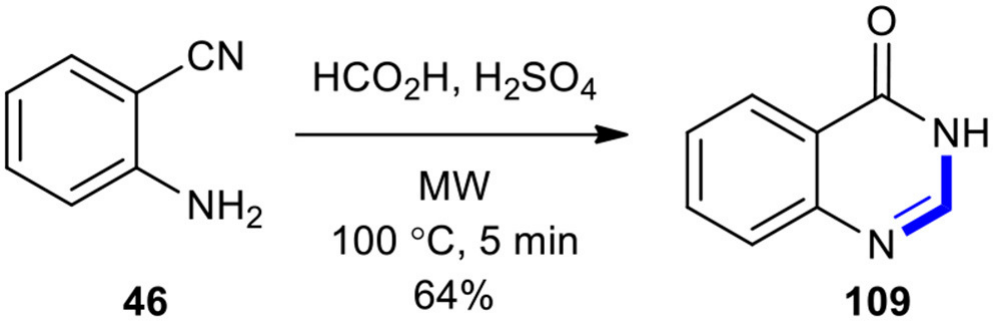
MW–assisted synthesis of **109** from **46** and formic acid.

The acid-catalyzed cyclization with formic acid was used to synthesize the 3*H*-quinazolin-4-one (**109**) by the employment of MWI (Saari et al., 2011). The product **109** was obtained in a 64% yield from the reaction of 2-cyanoaniline (**46**) with formic acid and sulfuric acid under MWI at 100°C for 5 min (Scheme 50).

#### TEAF

**Scheme 51:**
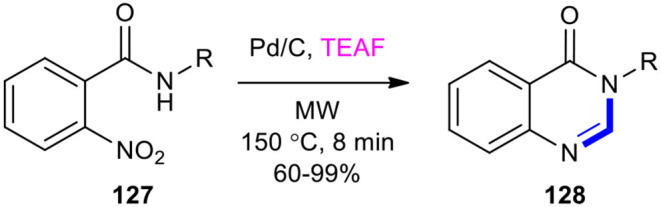
MW–assisted Pd-CTH/condensation cascade of **127** and TEAF to afford **128**.

Triethylamine–formic acid mixture (TEAF) has been used as a substrate for transition metal-catalyzed transfer hydrogenation and condensation with *o*-nitrobenzamides to produce the quinazolinones in a single-pot operation. For example, a highly efficient one-pot method for the synthesis of quinazolin-4(3*H*)-one derivatives **128** was developed using the Pd-catalyzed transfer hydrogenation (CTH)/condensation cascade of *o*-nitrobenzamides **127** and azeotropic TEAF under MWI at 150°C for 8 min (Scheme 51) (Zhu et al., 2015). In this reaction, TEAF played a dual role as a good hydrogen source for the CTH reduction and as a source of mono-carbon for the subsequent cyclocondensation.

#### Benzyl Alcohols

**Scheme 52:**
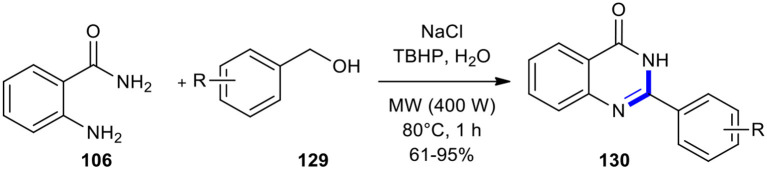
MW–assisted synthesis of **130** from **106** and **129** using NaCl and TBHP.

Benzyl alcohols have also been condensed with *o*-aminobenzamide derivatives under MW conditions to afford quinazolinones. A new water-assisted strategy to quinazolinones **130** was developed through the reaction of *o*-aminobenzamides (**106**) with benzyl alcohols **129** using sodium chloride as a salting-out agent and *tert*-butyl hydroperoxide (TBHP) as an oxidant under metal-, ligand-, base-free and MW conditions (Scheme 52) (Dandia et al., 2018). The production of **130** was examined under MWI and conventional heating conditions. The superior salting-out effect of sodium chloride was observed when the reaction was performed under MW conditions, compared to the conventional method. Among varying MWI power (300, 400, and 500 W) and temperatures, the 400 W power at 80°C gave the best result to carry out the maximum conversion to the expected product.

#### Thioamides, Isothiocyanates, and Potassium Isopropyldithiocarbonate

**Scheme 53:**
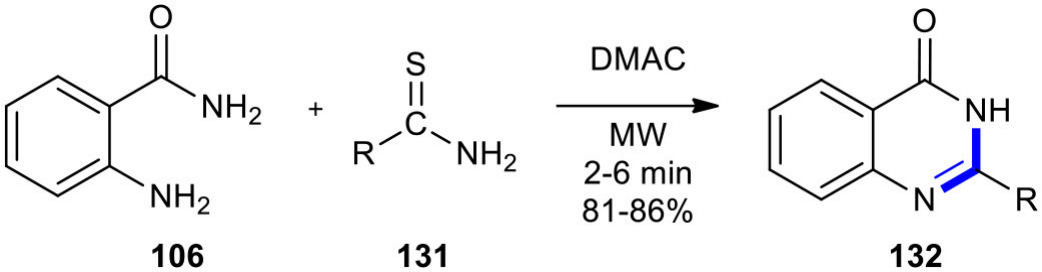
MW–assisted highly accelerated Niementowski reaction of **106** and **131** to afford **132**.

Thioamides, isothiocyanates, and potassium isopropyldithiocarbonate have also been employed as reactants to effect cyclocondensation of anthranilamide derivatives to afford quinazolinones. Khajavi et al. previously employed the thioamides for the highly accelerated Niementowski reaction under MWI (Khajavi et al., 1998). They condensed anthranilic acid (**106**) with thioamides (thiobenzamide or thioacetamide) **131** in DMAC using a house-hold unmodified microwave oven to generate 2-substituted-4-(3*H*)-quinazolinones **132**. The latter were obtained through a simple work-up procedure (Scheme 53). The conventionally thermalized approach for this reaction needed longer reaction times and resulted in the formation of **132** in lower yields.

**Scheme 54:**
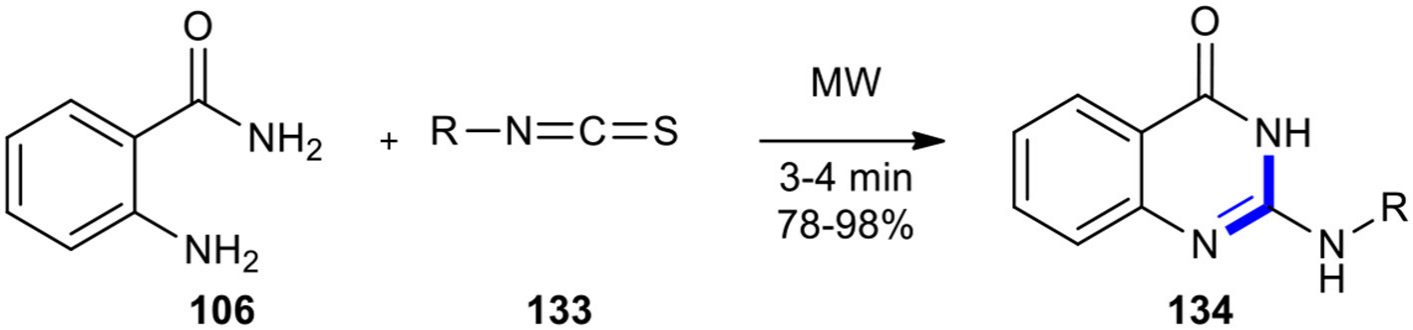
MW–assisted Niementowski reaction of **106** and **133** to afford **134**.

Tavallaii et al. previously reacted 2-aminobenzamide (**106**) with isothiocyanates (or isocyanates) **133** to access 2-(alkylamino) and 2-(arylamino)-4(3*H*) quinazolinones **134** (Tavalaie et al., 2007). The production of target molecules **134** was completed in high yields (78–98%) by applying MWI under solvent-free conditions, providing the best yields in a faster time in comparison to the conventional heating methods (Scheme 54).

**Scheme 55:**
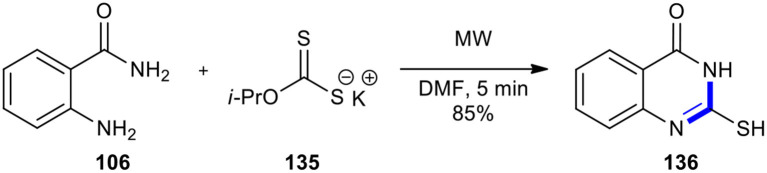
MW–assisted Niementowski reaction of **106** and **135** to afford **136**.

Kumar and Dubey in 2012 described a protocol which provided the safe, simple, rapid, inexpensive, and environmentally friendly process to 2-mercapto-quinazolinone (**136**) *via* the treatment of anthranilamide (**106**) as a bifunctional molecule with potassium isopropyldithiocarbonate (**135**) in a minimum quantity of DMF as solvent under MWI (Scheme 55) (Kumar and Dubey, 2012). While the reaction took 5 hours under reflux conditions, it lasted 5 min under MWI. Owing to high stability at higher temperatures and staying at room temperature for months, isopropyldithiocarbonate was used as a stable reagent in this method. These reaction conditions could also be applied to the transformation of various 1,2-bifunctional molecules to afford mercapto derivatives of benzimidazoles, benzothiazole and benzoxazole, and oxadiazoles.

#### Aliphatic and Aromatic Amines

**Scheme 56:**
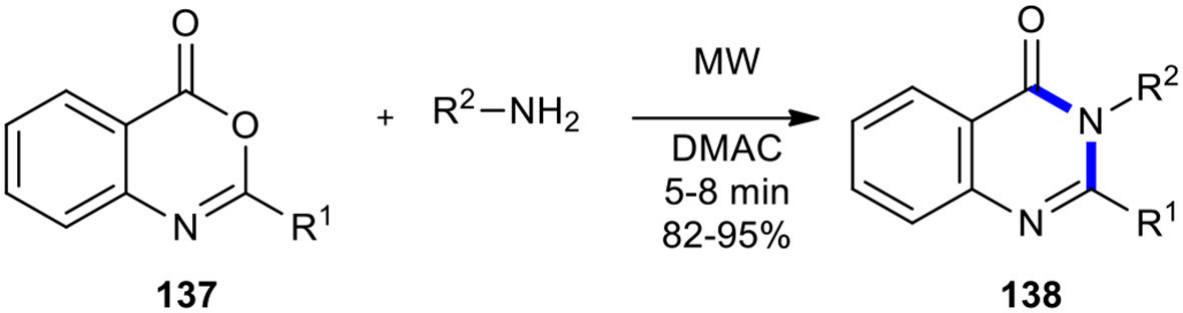
MW–assisted synthesis of **138** from **137** and amines in DMAC.

Benzoxazinones and their open-chain derivatives have also been employed as substrates for the synthesis of quinazolinones under MW conditions. For example, Khajavi et al. reported an efficient and practical synthesis of a number of variously substituted 4(3*H*)-quinazolinones **138** (Khajavi et al., 1999b). This transformation included the MWI in the combination of benzoxazinones **137** with amines in DMAC, enabling efficient access to this important class of heterocycles (Scheme 56). The MW–assisted chemistry provided increased product yields and shortened reaction times.

**Scheme 57:**
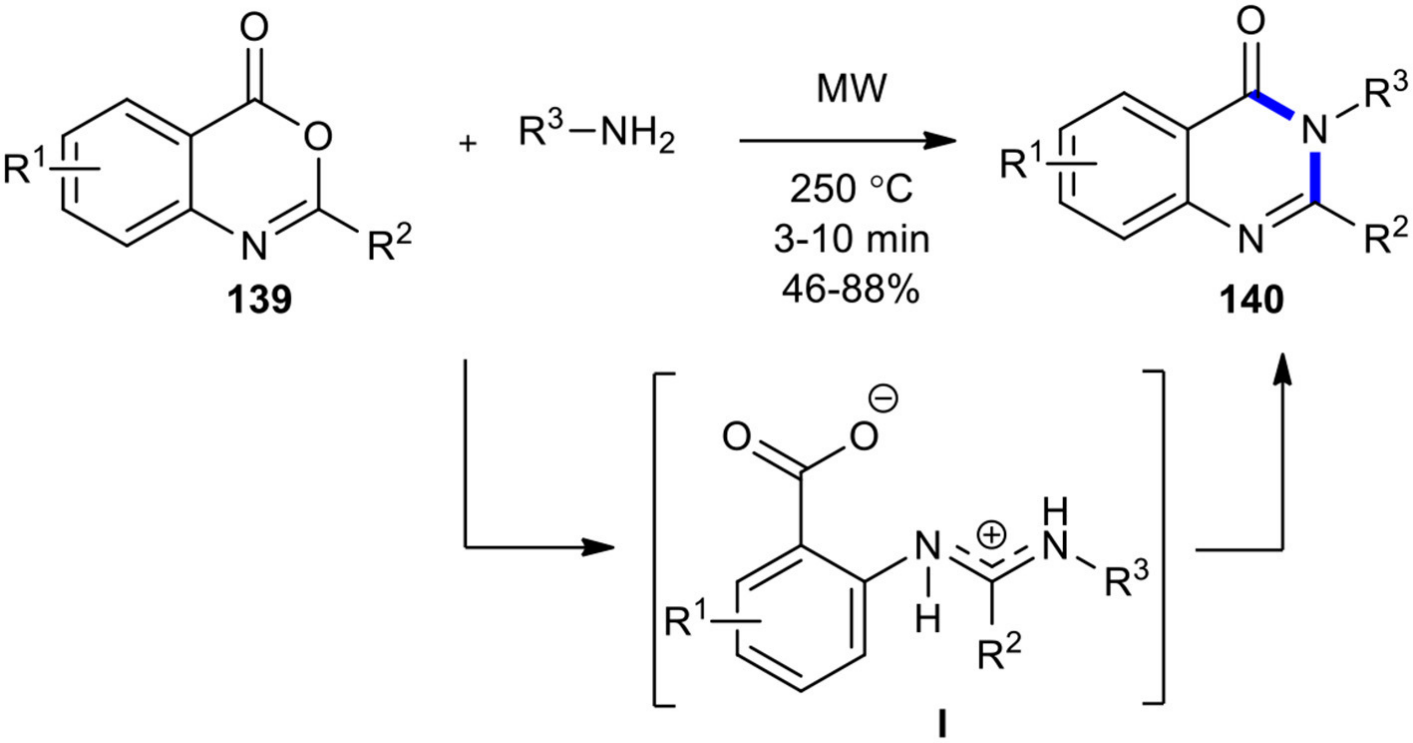
MW–assisted synthesis of **140** from **139** and amines.

2,3-Disubstituted 3*H*-quinazolin-4-ones **140** with a broad chemistry scope were synthesized by Liu's research group by employing MWI on benzoxazinones **139** with amines (Liu et al., 2005b). Benzoxazinones **139** prepared by the condensation of anthranilic acids and either acyl chlorides or carboxylic acids using P(PhO)_3_ were, in turn, condensed with amines to provide the transient amidine salt species (**I)**. This intermediate rapidly cyclized under the optimized MW conditions at 250°C in 3–10 min, yielding the expected 2,3-disubstituted 3*H*-quinazolin-4-ones **140** (Scheme 57).

**Scheme 58:**
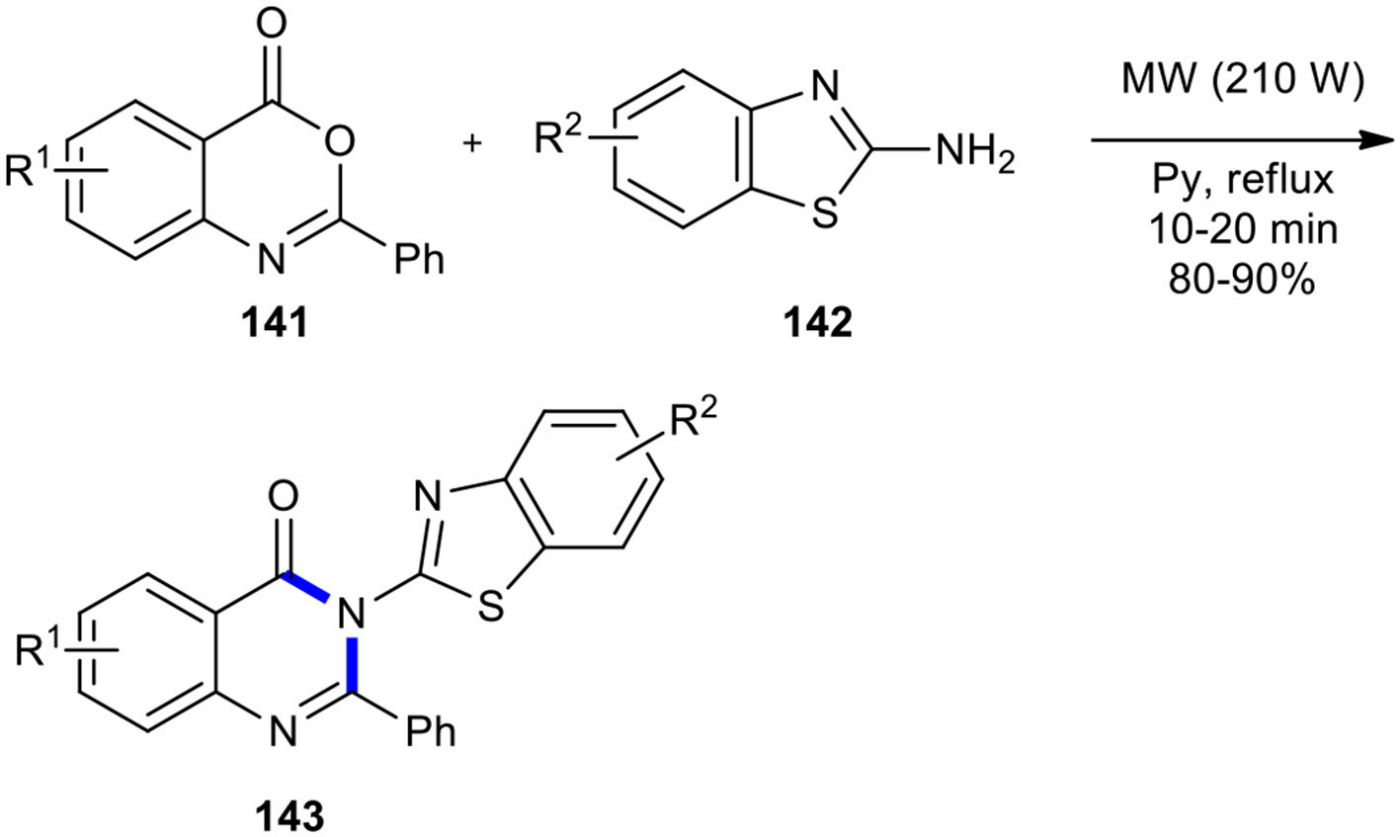
MW-accelerated solution-phase synthesis of **143** from **141** and **142**.

Laddha and Bhatnagar employed the MW-accelerated solution-phase method to 6,8-disubstituted 2-phenyl-3-(benzothiazol-2-yl)-4-[3*H*]-quinazolinones **143**, bearing various substituents on the benzothiazole ring (Laddha and Bhatnagar, 2008). The synthesis involved the cyclocondensation of 2-phenyl-4*H*-benzo[*d*][1,3]oxazine-4-ones **141** with 2-aminobenzothiazoles **142** in dry pyridine under MWI at 210 W and reflux temperature (Scheme 58). This procedure proceeded very cleanly and formed products in a fast and easy work-up procedure without any traces of side products, presenting the advantage of a high rate and better yields than the conventional procedure.

**Scheme 59:**
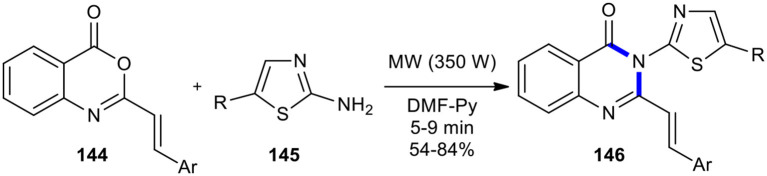
MW–assisted synthesis of **146** from **144** and **145**.

Various 2-styryl benzoxazinone derivatives **144** were utilized with 2-aminothiazoles **145** using pyridine-DMF as a co-solvent under MWI in the synthesis of newer 3-thiazole substituted 2-styryl-4(3*H*)-quinazolinones **146** (Jagani et al., 2012). The products **146** were obtained in good yields within an appropriate time of MWI at 350 W (Scheme 59). It is interesting to know that the reaction either in pyridine as a solvent proceeded sluggishly or in DMF reduced yields.

**Scheme 60:**
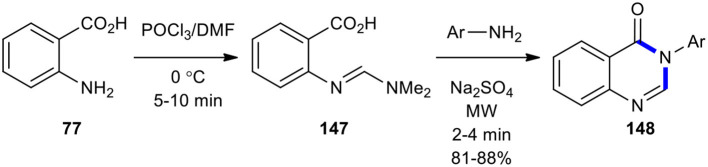
MW–assisted synthesis of **148** from **147** and anilines.

Priya et al. prepared compound **147** by the reaction of anthranilic acid (**77**) with Vilsmeier reagent (DMF/POCl_3_) (Heravi et al., 2018). They then reacted **147** with differently substituted anilines under MW for 2–4 min to synthesize 4-(3*H*)-quinazolinone derivatives **148** in good yields (Scheme 60) (Priya et al., 2011). The use of MWI in this reaction shows its value in providing increased yields in short reaction times.

**Scheme 61:**
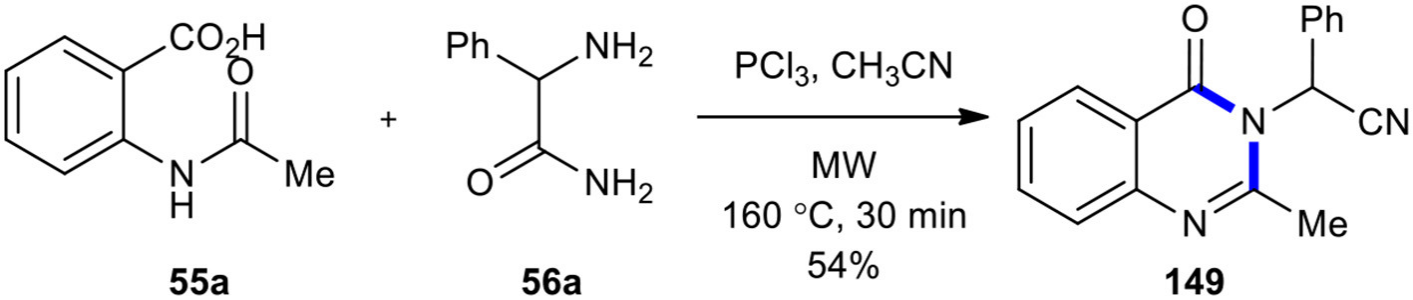
MW–assisted synthesis of **149** from **55a** and **56a** using PCl_3_.

In 1946, Grimmel's research group reported the synthesis of quinazolinones through the treatment of *N*-acetylanthranilic acids with aromatic amines using the condensing agents including POCl_3_, phosphorus trichloride (PCl_3_), or thionyl chloride(SOCl_2_) in toluene or xylene (Grimmel et al., 1946). This method has been frequently used to synthesize a wide range of quinazolinone derivatives (Wolfe et al., 1990; van Zyl, 2001; Bhatti and Seshadri, 2004; Storelli et al., 2005; Giri et al., 2009). In 2009, MWI was used to synthesize 2-(2-methyl-4-oxoquinazolin-3(4*H*)-yl)-2-phenylacetonitrile (**149**) (Li et al., 2009). When *N*-acetylanthranilic acid (**55a**) was reacted with the amino group of 2-amino-2-phenylacetamide (**56a**) using PCl_3_ as a condensing reagent in acetonitrile under MWI at 160°C for 30 min, quinazolinone was formed with concomitant carboxamide dehydration to give 2-(2-methyl-4-oxoquinazolin-3(4*H*)-yl)-2-phenylacetonitrile (**149**) in a 54% yield (Scheme 61).

**Scheme 62:**
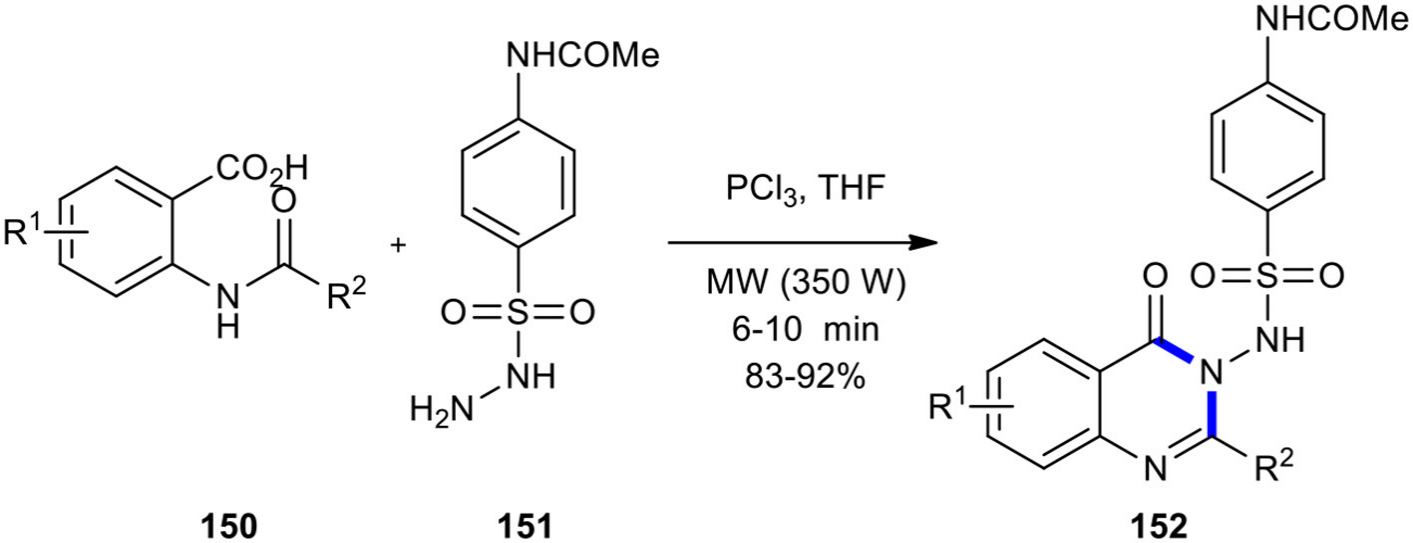
MW–assisted synthesis of **152** from **150** and **151** using PCl_3_.

Jagani et al. (2011) optimized Grimmel's method (Grimmel et al., 1946) using MWI to construct *N*-(4-(*N*-(4-oxo-2-methyl/aryl-substituted quinazolin-3(4*H*)-yl)sulfamoyl)phenyl)acetamides **152**, allowing the improvement of rate and yields. The quinazolinone products **152** were synthesized by the reaction between *N*-acetylanthranilic acids **150** and 4-acetamidobenzenesulfonyl hydrazide (**151**) in the presence of an amount of PCl_3_ in THF as a solvent under MW conditions (350 W) (Scheme 62).

**Scheme 63:**
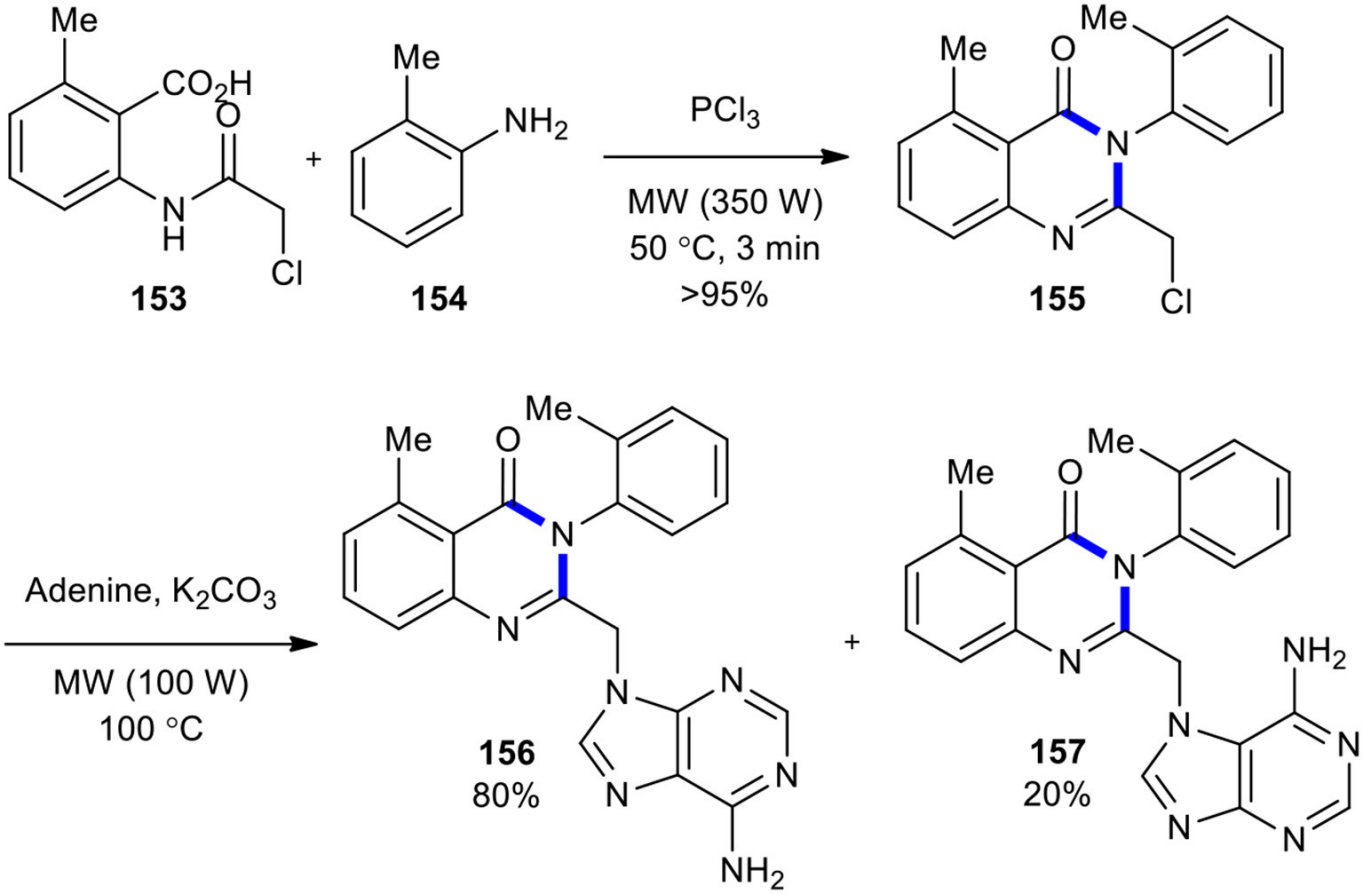
MW–assisted synthesis of **155** and further transformation into **156** and **157**.

A protocol that involved an environmentally friendly MW–assisted cyclization reaction to construct 2-(chloromethyl)-5-methyl-3-(o-tolyl)quinazolin-4(3*H*)-one (**155**) toward the medicinally important purine quinazolinone scaffold was devised and presented (Sawant et al., 2012). The reaction of compound **153** with 2-methyl aniline (**154**) and PCl_3_ as a cyclizing agent under MW conditions (350 W) at 50°C for 3 min yielded 2-(chloromethyl)-5-methyl-3-*o*-tolylquinazolin-4(3*H*)-one (**155**) in excellent yield (>95%). Compound **155** without purification was subjected to a coupling reaction with adenine to give the expected molecules **156** and **157** (Scheme 63).

### Three-Component Reaction

**Scheme 64:**
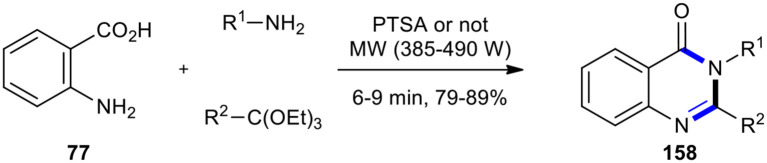
MW–assisted MCR of **77**, amine, and ortho-esters using PTSA to produce **158**.

MCR, as one of the most straightforward strategies, has been extensively employed under MWI for the synthesis of quinazolinone derivatives. For example, the preparation of substituted quinazolin-4(3*H*)-one derivatives **158** by the one-pot three-component cyclocondensation of anthranilic acid (**77**) with an amine and formic acid (or ortho-esters) under MWI was reported by Rad-Moghadam and Khajavi (Scheme 64) (Rad-Moghadam and Khajavi, 1998). Formic acid and amines were used in place of amides or amidines utilized in the Niementowski reaction, providing 3-substituted-4(3*H*)quinazolinones **158** instead of the resulting 2-substituted derivative. The reaction was completed in a few minutes in the absence of solvent or any dehydrating agents. As a result, the reaction with ortho-esters required a catalytic amount of PTSA. The reaction occurred within a much reduced time under MWI with respect to the conventional heating procedure (Leiby, 1985).

**Scheme 65:**
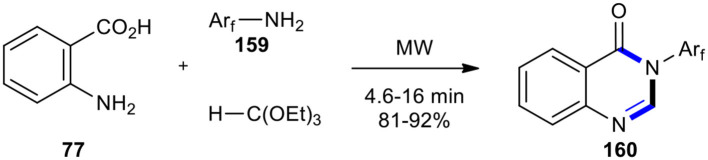
MW–assisted MCR of **77, 159**, and triethoxymethane to create **160**.

A modification of the above method, which is environmentally friendly, involved the synthesis of fluorine-containing 4(3*H*)-quinazolinones **160** using fluorinated anilines **159** in the absence of a solvent and catalysts (Scheme 65) (Wenli et al., 2011).

**Scheme 66:**
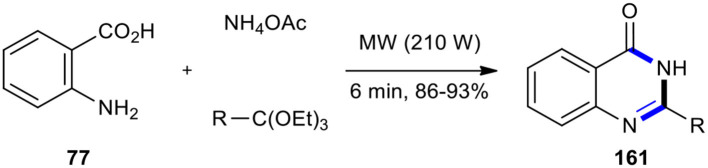
MW–assisted MCR of **77**, ammonium acetate, and ortho-esters to synthesize **161**.

The demand for increasingly clean and efficient chemical synthesis of 4-(3*H*)-quinazolinone from anthranilic acid, amines, and ortho-esters or formic acid under MW conditions paved the way for the use of Yb(OTf)_3_ (Wang et al., 2003), NaHSO_4_-SiO_2_ (Das and Banerjee, 2004), silica gel/FeCl_3_ (Chari et al., 2006), La(NO_3_)_3_, Bi(TFA)_3_-[nbp]FeCl_4_ (Khosropour et al., 2006), Nafion-H (perfluorinated resin supported sulfonic acid) (Lingaiah et al., 2006), H-Y-zeolites (Bakavoli et al., 2007), Keggin-type heteropolyacids (HPAs) (Ighilahriz et al., 2008), and silica sulfuric acid (SSA) (Koroji et al., 2018) as solid supported catalysts and PTSA (Narasimhulu et al., 2006) and SnCl_4_ (Oskooie et al., 2007) as catalysts. It is noteworthy that the use of ammonium acetate as a source of amine in the condensation with anthranilic acid (**77**) and ortho-esters under MWI in the absence of a solvent yielded the corresponding 2-substituted quinazolin-4(3*H*)-ones **161** in a few minutes (Scheme 66) (Rad-Moghadam and Mohseni, 2003).

**Scheme 67:**
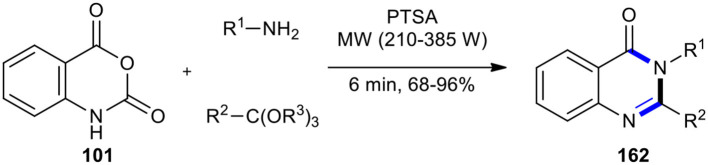
MW–assisted MCR of **101**, amines, and ortho-esters using PTSA to afford **162**.

One of the methods for the synthesis of substituted (3*H*)-quinazolin-4-one derivatives involved a one-pot three-component condensation of isatoic anhydride (**101**) with amines and ortho-esters. In 2014, Dabiri et al. developed this method by employing MWI in the presence of PTSA as a catalyst, resulting in the formation of 2,3-disubstituted 4(3*H*)-quinazolinones **162** in good to excellent yields (Scheme 67) (Dabiri et al., 2004a). They also compared the resulting products synthesized under the two conditions of MW and classical heating, which indicated the superiority of MW conditions. The method offered several advantages including a cleaner reaction, short reaction times, a high yield of products, and an easy experimental work-up procedure.

**Scheme 68:**
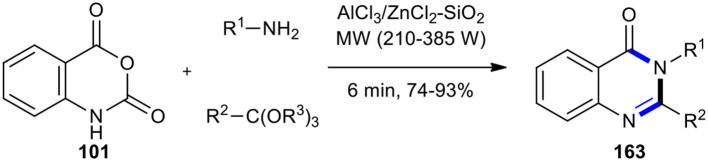
MW–assisted MCR of **101**, amines, and ortho-esters using AlCl_3_/ZnCl_2_-SiO_2_.

Later, the application of the AlCl_3_/ZnCl_2_-SiO_2_ Lewis acid system instead of PTSA under both MWI and conventional heating methods was studied by the same group. Disubstituted (3*H*)-quinazolin-4-ones **163** were synthesized in good yields under conventional conditions, although slightly higher yields were obtained under MWI (Scheme 68) (Dabiri et al., 2005). It should be noted that the work-up of products under both conditions was very easy since they were washed with hot ethanol and then filtrated and recrystallized.

**Scheme 69:**
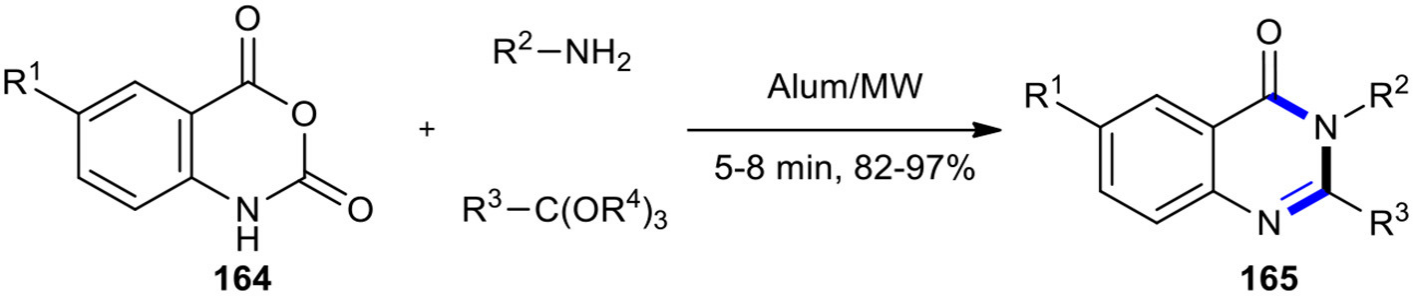
MW–assisted MCR of **164**, amines, and ortho-esters using Alum to afford **165**.

KAl(SO_4_)_2_∙12H_2_O (Alum) is an inexpensive and nontoxic reagent which is very soluble in water and is also recyclable. Its ability as an effective catalyst was demonstrated in the MW–assisted synthesis of 2-alkyl and 2-aryl-4(3*H*)-quinazolinones. Twenty 2,3-disubstituted-4(3*H*)-quinazolinones **165** were synthesized through a three-component reaction between isatoic anhydrides **164**, ortho-esters, and amines (Scheme 69) (Mohammadi and Sadat Hossini, 2011). The high yield of products and simple experimental work-up procedures are the salient features of this method.

**Scheme 70:**
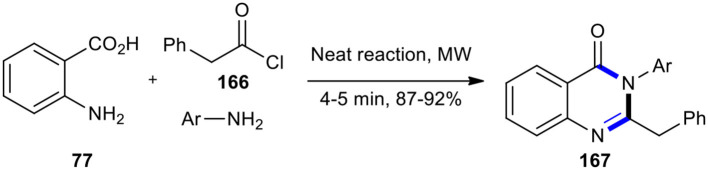
MW–assisted MCR of **77, 166**, and anilines to afford **167**.

The synthesis of fluorinated 2,3-disubstituted quinazolin-4(3*H*)-ones **167** was reported by Dandia et al. by developing a “green chemistry procedure” using a neat one-pot three-component cyclocondensation under MW conditions (Dandia et al., 2004). Treatment of anthranilic acid (**77**) with phenyl acetyl chloride (**166**) and substituted anilines in the microwave oven at 640 W for 4–5 min without using any solvent, dehydrating agent, or support produced the target molecules **167** (Scheme 70). The MW chemistry in this reaction allowed significant rate enhancement and good to excellent yields, compared to the conventional procedure.

**Scheme 71:**
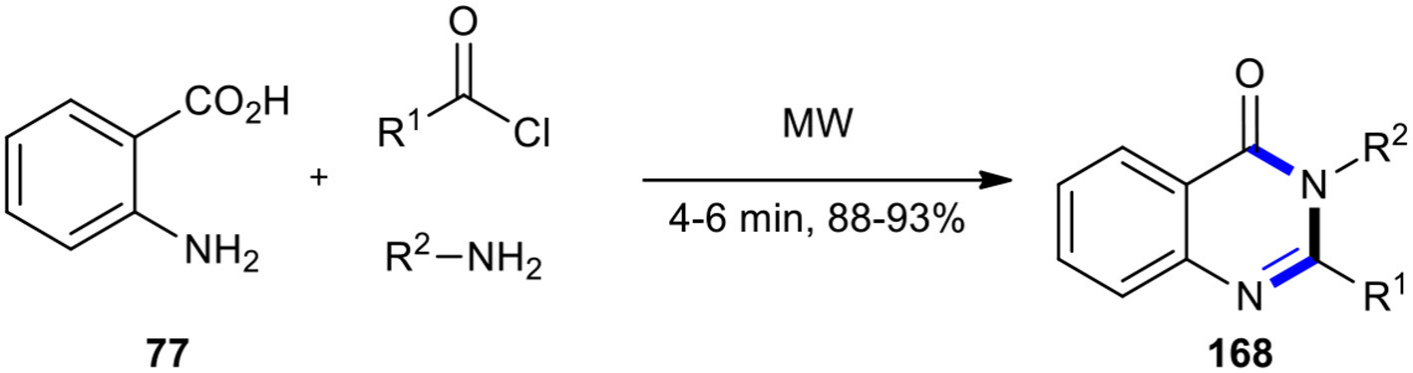
MW–assisted MCR of **77**, acyl chlorides, and amines to afford **168**.

An approach that utilized anthranilic acid and formamide to the synthesis of quinazolin-4(3*H*)-one core was improved by using the neat MW–assisted three-component reaction of anthranilic acid (**77**), acyl chlorides, and amines (Dandia et al., 2005). The reaction was completed in a shorter time with a facile work-up procedure, making it a general protocol to produce the expected molecules **168** with a range of acid chlorides and amines. Using MWI at 640 watts, the reaction between anthranilic acid, acid chlorides, and amines furnished 2-benzyl-3-aryl-quinazolin-4-[3*H*]-ones **168** in high yields and reduced the time (Scheme 71).

**Scheme 72:**
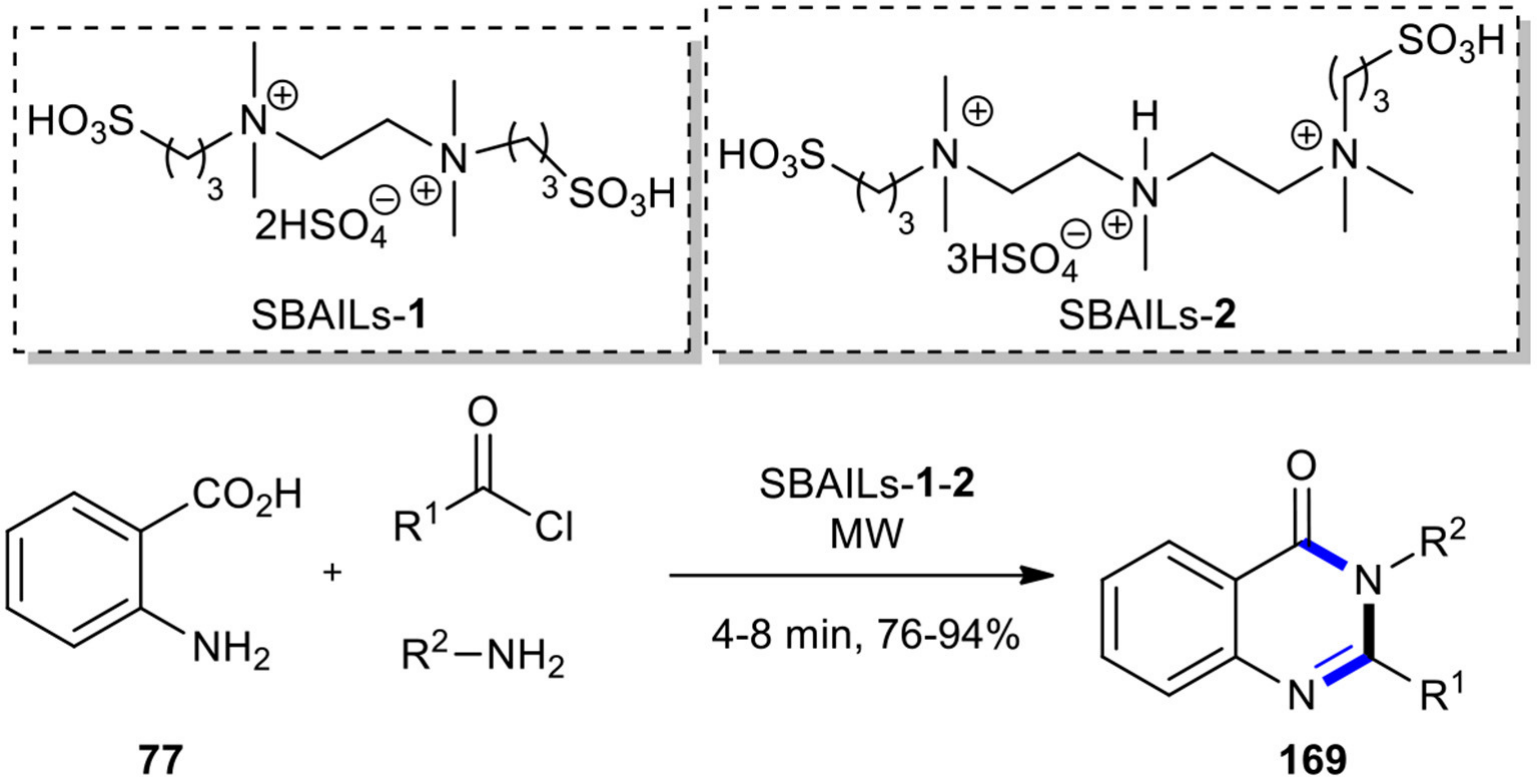
MW–assisted MCR of **77**, acyl chlorides, and amines using SBAILs-**1** and **2**.

After a decade, the same procedure in the presence of two long-chain double SO_3_H-functionalized acidic ionic liquids, SBAILs-**1** and **2**, as reusable catalysts, was reported (Scheme 72) (Li et al., 2015). Although the authors have expanded the production of 2,3-disubstituted 4(3*H*)-quinazolinone derivatives **169**, one product formed (R^1^ = Ph, R^2^ = 4-Cl-C_6_H_5_), which was produced from the previous method (Dandia et al., 2005), with a lesser yield (Li et al., 2015).

**Scheme 73:**
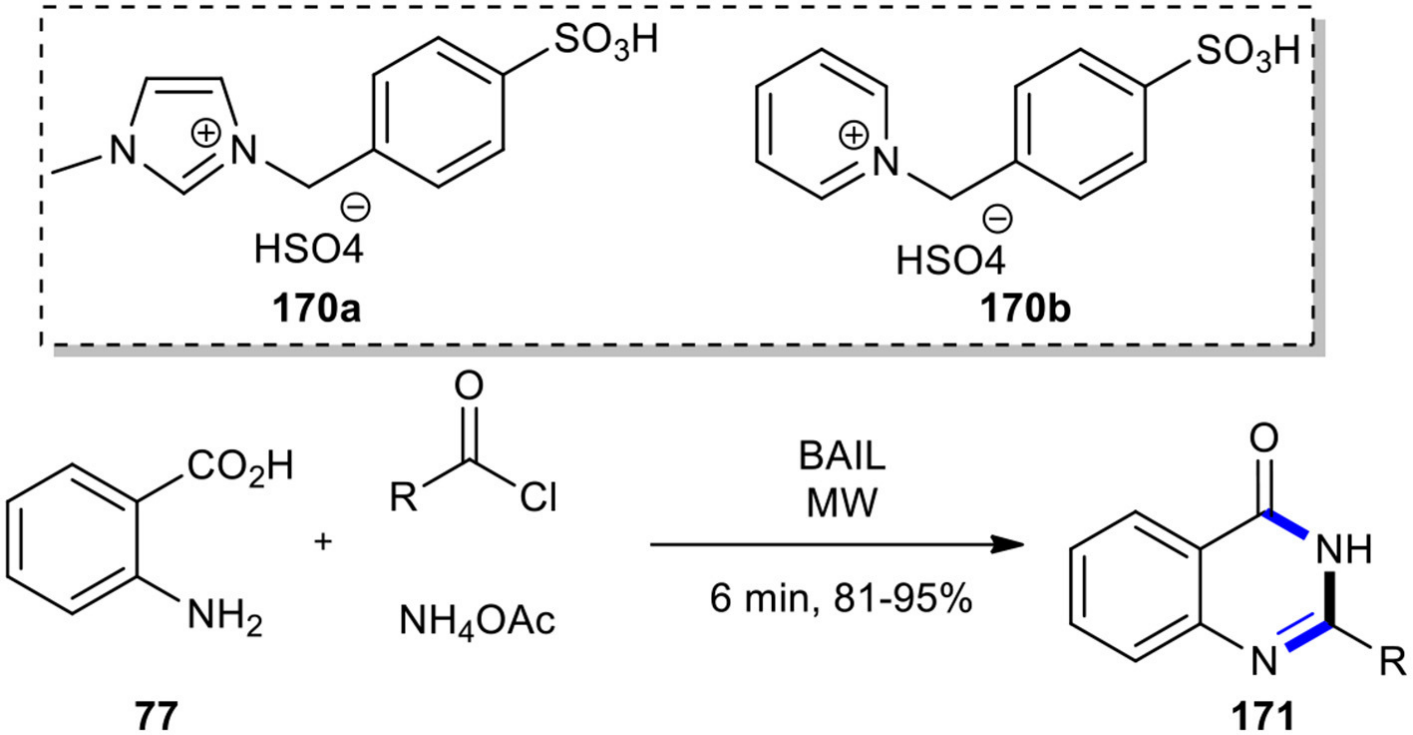
MW–assisted MCR of **77**, acyl chlorides, and NH_4_OAc using BAIL to afford **171**.

Two new Brønsted acidic ionic liquids, including 1-(4-sulfonic)-benzyl-3-methylimidazolium hydrogen sulfate (**170a**) and *N*-(4-sulfonic)-benzyl-pyridium hydrogen sulfate (**170b**), were utilized as catalyst and reaction medium, respectively, to lead to the synthesis of 2-substituted-4(3*H*)-quinazolinones **171** in satisfactory yields (Li et al., 2010). The reaction proceeded through a one-pot three-component reaction under MW conditions for 6 min, starting from anthranilic acid (**77**), acyl chlorides, and ammonium acetate (Scheme 73).

**Scheme 74:**
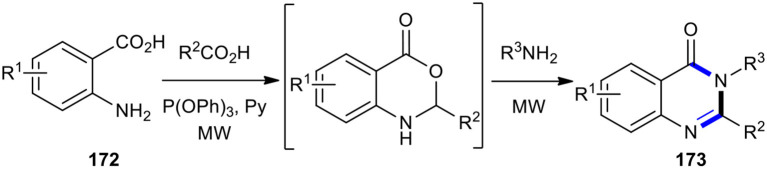
MW–assisted MCR of **172**, carboxylic acids, and amines to afford **173**.

Liu et al. developed a practical and efficient protocol that employed an MW–assisted one-pot three-component reaction for the synthesis of various 2,3-disubstituted 3*H*-quinazolin-4-ones **173**, starting from anthranilic acids **172**, carboxylic acids, and amines (Scheme 74) (Liu et al., 2005b). The use of MW–assisted chemistry increased the product yields and shortened the reaction times.

**Scheme 75:**
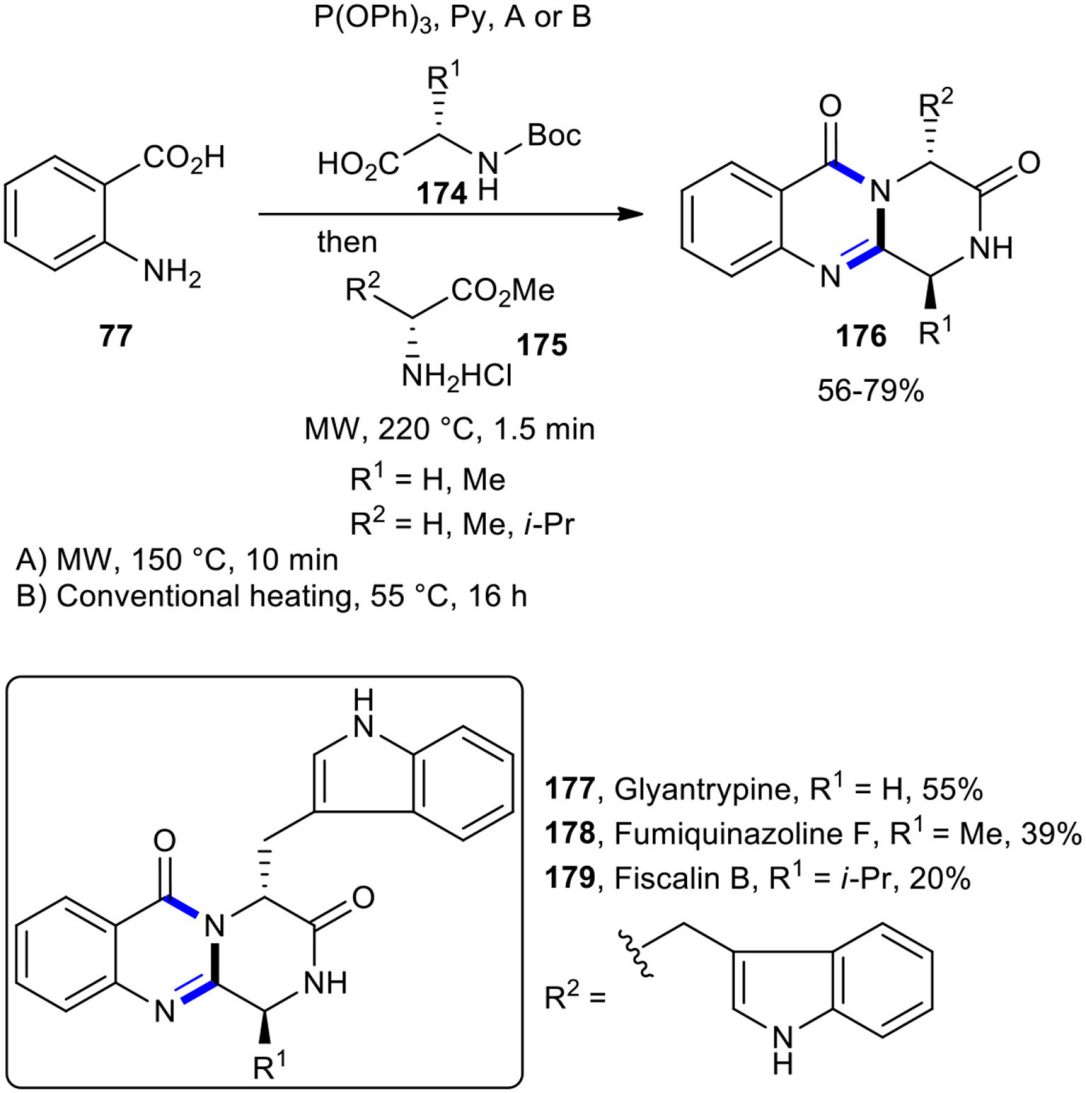
The synthesis of **176** and total synthesis of **177, 178**, and **179** under MWI.

This MW–assisted one-pot methodology could be adapted for the synthesis of 4-quinazoline-3,6-diones **176** and also for the total synthesis of a number of natural products containing core, accommodating an expanded array of carboxylic acids and amines (Liu et al., 2005c). Using selected Boc-amino acids and amino acid esters under MWI, total synthesis of the alkaloids glyantrypine (**177**), fumiquinazoline F (**178**), and fiscalin B (**179**) was accomplished in overall yields of 55, 39, and 20%, respectively (Scheme 75).

**Scheme 76:**
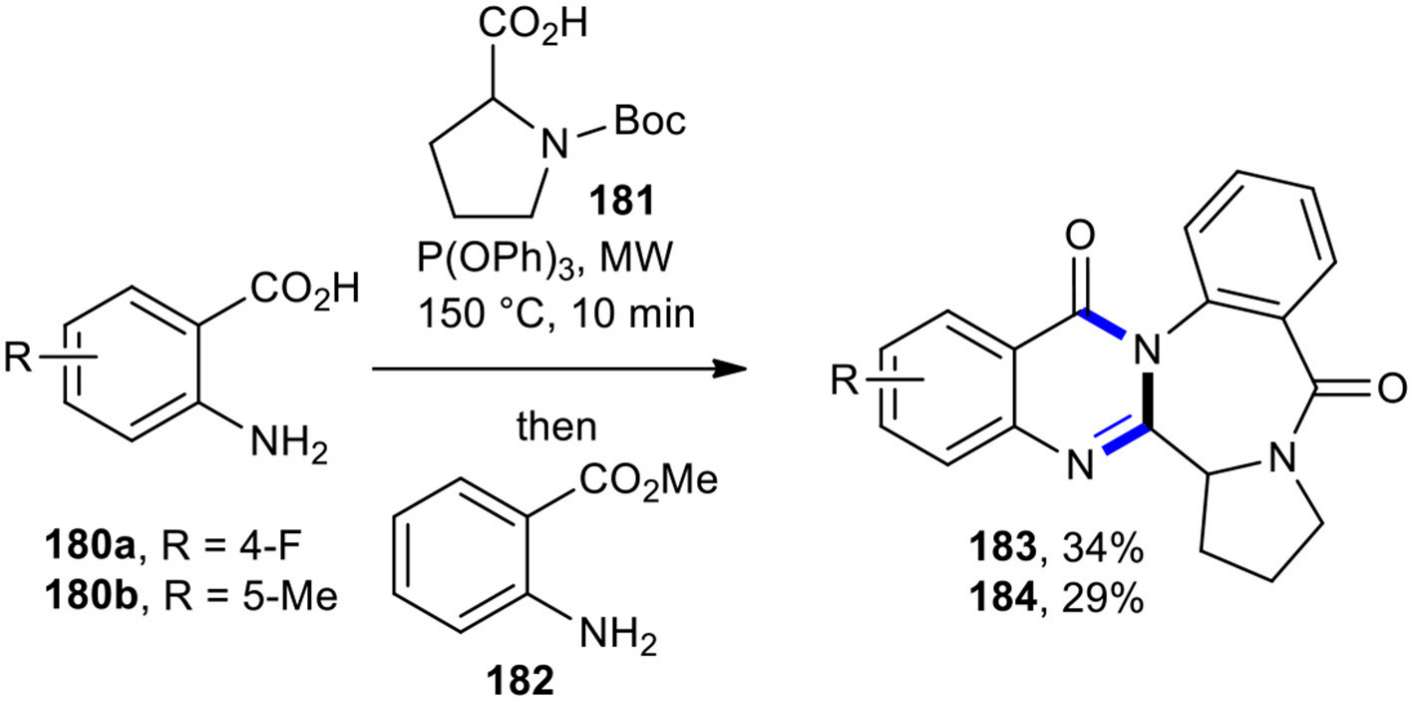
MW–assisted synthesis of circumdatin E analogs **183** and **184**.

Liu's research group synthesized circumdatin E analogs **183** and **184** by using MW–assisted one-pot three-component sequential reactions (Liu et al., 2005a). The subjection of anthranilic acids **180a** and **180b** to MWI three-component reaction with *N*-Boc-proline (**181**) using P(OPh)_3_ reagent in pyridine at 150°C in 10 min, followed by the addition of methyl anthranilate (**182**) and further conditions (MW, 230°C, 15 min), resulted in the formation of the expected compounds **183** and **184** as analogs of circumdatin E in 34 and 29% yields, respectively (Scheme 76).

**Scheme 77:**
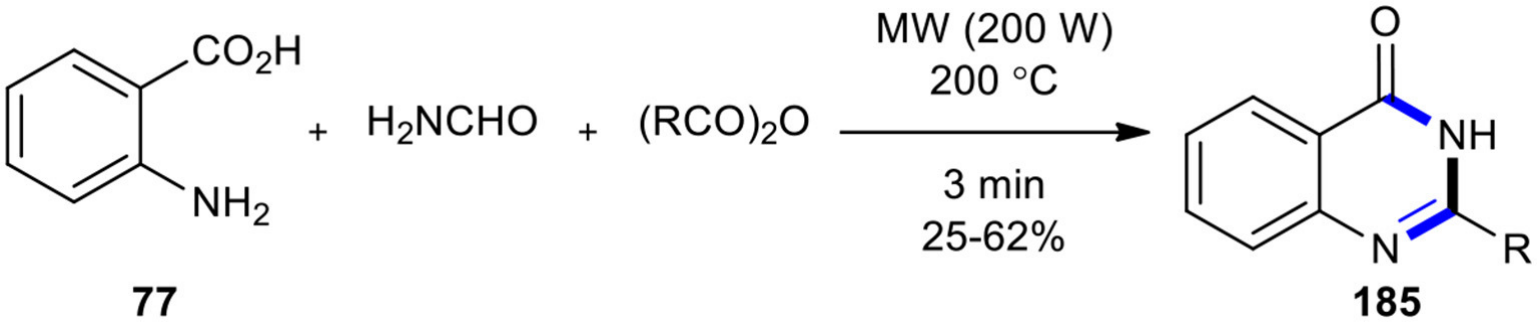
MW–assisted MCR of **77**, carboxylic anhydride, and formamide to afford **185**.

Wan et al. reported Pd-catalyzed aminocarbonylations of aryl halides by the employment of MW-accelerated decomposition of formamide to ammonia (NH_3_) and carbon monoxide (CO) (Wan et al., 2003). After a couple of years, Nouira et al. achieved this reaction by using NH_3_ as the sole synthon for introducing a nitrogen atom in a heterocyclic ring *via* MW–assisted three-component reactions with anthranilic acid (**77**) and carboxylic anhydride, leading to the fast and safe synthesis of some quinazolin-4-one derivatives **185** (Scheme 77) (Nouira et al., 2008). Their work confirmed that the use of MWI may lead to different behaviors of reactants, depending on parameters such as power input, reached temperature, and pressure in the vials. Using modern MW technology, full control and fine-tuning of these parameters can be achieved.

**Scheme 78:**
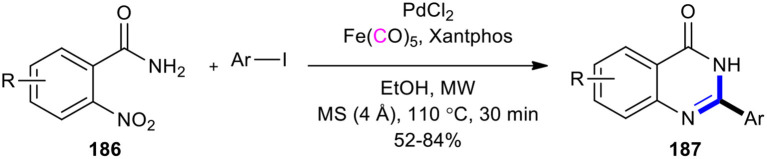
MW–assisted iron pentacarbonyl mediated carbonylation of aryl iodides.

Lin et al. discovered a rapid, direct, and practical method for the construction of a quinazolinone scaffold by using iron pentacarbonyl (Fe(CO)_5_) as a reducing agent and CO source under MW and base-free conditions (Lin et al., 2017). The process involved the Pd/Fe(CO)_5_-catalyzed reduction of *o-*nitrobenzamides to *o-*aminobenzamides, followed by reductive carbonylation of aryl iodides into amide intermediates and subsequent intramolecular ring closure. To access 2-substituted quinazolinones **187**, various *o-*nitrobenzamides **186** and aryl iodides using various palladium catalysts, ligands, and solvents were employed. The best reaction conditions were found to use Pd(Cl)_2_ as a catalyst, xantphos as a ligand, and ethanol as a solvent at 110°C in 30 min under MWI in good yields (Scheme 78).

## Conclusions

In this review, we highlighted the application of MWI in the synthesis of quinazolines and quinazolinones. Routes and strategies used here have opened a gateway to the wider applications of MW chemistry in both academia and industry. Several quinazoline and quinazolinone derivatives were provided with a broad substrate scope in a variety of MW systems, e.g. the domestic microwave oven. Many reactions were examined under both MW and conventional heating conditions in which the superiority of MW conditions was demonstrated. MWI acted as a powerful technique that could reduce the reaction times (more often a few minutes), enhance the yield of the products, increase the purity of the resulting product, and decrease the usage of organic solvents and by-products more than the conventional heating procedure. The salient features of MW reactions in these syntheses include environmental friendliness, more economic value, good atom economy, and simple experimental work-up procedure. Due to previous features and the lack of a need for solvents, reagents, and ligands in many cases, MW technology was able to promote the principles of green chemistry. It should be noted that the reactions may occur under the conditions of the MW heating or at room temperature, indicating the significant effect of temperature on the reaction. In many reactions, it is shown that focused MWI with proper control of power and temperature is more important and efficient than multimode MWI or conventional heating sources. Most of the strategies developed for the synthesis of quinazoline and quinazolinone derivatives under MWI were successfully achieved under metal-free conditions. The merits of metal-free reactions involve the absence of a costly metal catalyst and supporting ligands, comparatively milder reaction conditions, insensitivity to moisture, and easy workup procedures. Few examples of synthesis of quinazoline and quinazolinone derivatives using transition metals such as zinc, iron, and copper were described.

## Author Contributions

MH has been invited by the editor, developed the idea, and drafted the presented manuscript and worked on it. LM presented the idea and wrote the manuscript. All authors contributed to the article and approved the submitted version.

## Conflict of Interest

The authors declare that the research was conducted in the absence of any commercial or financial relationships that could be construed as a potential conflict of interest.
